# Maternal dendritic cells influence fetal allograft response following murine in-utero hematopoietic stem cell transplantation

**DOI:** 10.1186/s13287-023-03366-9

**Published:** 2023-05-24

**Authors:** Karthikeyan Kandasamy, Nuryanti Binti Johana, Lay Geok Tan, Yvonne Tan, Julie Su Li Yeo, Nur Nazneen Binte Yusof, Zhihui Li, Jiayu Koh, Florent Ginhoux, Jerry K. Y. Chan, Mahesh Choolani, Citra N. Z. Mattar

**Affiliations:** 1 Experimental Fetal Medicine Group, Department of Obstetrics and Gynaecology, Yong Loo Lin School of Medicine, National University of Singapore, 1E Kent Ridge Road, Singapore, 119228 Singapore; 2 Department of Obstetrics and Gynaecology, National University Health System, National University Hospital, Singapore, Singapore; 3 Reproductive Medicine, KK Women’s and Children’s Hospital, Singapore, Singapore; 4 Cancer and Stem Cell Biology Program, Duke-NUS Graduate Medical School, Singapore, Singapore; 5 Genome Research Informatics and Data Science Platform, Genome Institute of Singapore, Agency for Science Technology and Research, Singapore, Singapore; 6 Singapore Immunology Network (SIgN), Agency for Science, Technology and Research (A*STAR), Singapore, Singapore; 7 Translational Immunology Institute, Singhealth/Duke-NUS Academic Medical Centre, The Academia, Singapore, Singapore; 8 Shanghai Institute of Immunology, Shanghai JiaoTong University School of Medicine, Shanghai, China

**Keywords:** Hematopoietic stem cells, In-utero transplantation, Fetal tolerance, Maternal microchimerism

## Abstract

**Background:**

Intrauterine hematopoietic stem cell transplantation (IUT), potentially curative in congenital haematological disease, is often inhibited by deleterious immune responses to donor cells resulting in subtherapeutic donor cell chimerism (DCC). Microchimerism of maternal immune cells (MMc) trafficked into transplanted recipients across the placenta may directly influence donor-specific alloresponsiveness, limiting DCC. We hypothesized that dendritic cells (DC) among trafficked MMc influence the development of tolerogenic or immunogenic responses towards donor cells, and investigated if maternal DC-depletion reduced recipient alloresponsiveness and enhanced DCC.

**Methods:**

Using transgenic CD11c.DTR (C57BL/6) female mice enabled transient maternal DC-depletion with a single dose of diphtheria toxin (DT). CD11c.DTR females and BALB/c males were cross-mated, producing hybrid pups. IUT was performed at E14 following maternal DT administration 24 h prior. Bone marrow-derived mononuclear cells were transplanted, obtained from semi-allogenic BALB/c (paternal-derived; pIUT), C57BL/6 (maternal-derived; mIUT), or fully allogenic (aIUT) C3H donor mice. Recipient F1 pups were analyzed for DCC, while maternal and IUT-recipient immune cell profile and reactivity were examined via mixed lymphocyte reactivity functional assays. T- and B-cell receptor repertoire diversity in maternal and recipient cells were examined following donor cell exposure.

**Results:**

DCC was highest and MMc was lowest following pIUT. In contrast, aIUT recipients had the lowest DCC and the highest MMc. In groups that were not DC-depleted, maternal cells trafficked post-IUT displayed reduced TCR & BCR clonotype diversity, while clonotype diversity was restored when dams were DC-depleted. Additionally, recipients displayed increased expression of regulatory T-cells and immune-inhibitory proteins, with reduced proinflammatory cytokine and donor-specific antibody production. DC-depletion did not impact initial donor chimerism. Postnatal transplantation without immunosuppression of paternal donor cells did not increase DCC in pIUT recipients; however there were no donor-specific antibody production or immune cell changes.

**Conclusions:**

Though maternal DC depletion did not improve DCC, we show for the first time that MMc influences donor-specific alloresponsiveness, possibly by expanding alloreactive clonotypes, and depleting maternal DC promotes and maintains acquired tolerance to donor cells independent of DCC, presenting a novel approach to enhancing donor cell tolerance following IUT. This may have value when planning repeat HSC transplantations to treat haemoglobinopathies.

**Supplementary Information:**

The online version contains supplementary material available at 10.1186/s13287-023-03366-9.

## Introduction

Intrauterine hematopoietic stem cell transplantation (IUT) has the potential to cure several congenital hematological disorders, with numerous advantages over conventional postnatal hematopoietic stem cell transplantation, particularly the avoidance of myeloablation and immunosuppression [1]. Clinical application of IUT has however been hampered by poor engraftment due to numerous engraftment barriers, of which fetal and maternal immune responses to transplanted cells are formidable examples, leading to loss of donor cell chimerism (DCC) by immunological clearance [2–5]. Active trafficking of maternal immune cells (maternal microchimerism, MMc) to the fetus occurs throughout pregnancy, and can persist for years after birth [6, 7]. Substantial increases in trafficked maternal leukocytes and alloantibodies into recipient fetuses follow intrauterine transplantation of stem cells [8, 9], infusion of gene therapy vectors [10], and in response to the invasive procedure itself [11], which in turn limits donor cell engraftment. Previously we demonstrated the selective trafficking of maternal CD4, CD8, CD19 and CD11c immune cells into fetuses accompanying IUT, and haploidentical donor cells derived from paternal bone marrow engrafted more efficiently than maternal donor cells [8]. This was associated with a more regulatory T cell (Treg) and less pro-immune and pro-inflammatory recipient immune profile. Maternal and fetal dendritic cells (DC) are important to both innate and adaptive immunity, and, being the most important antigen-presenting cells for naïve T cells [12], mediate antigen-specific tolerance via altered expression of costimulatory molecules and cytokines [13–15]. DC can produce immunogenic or tolerogenic responses by altering the balance of Th1/Th17/Th2 cells as dictated by the specific microenvironment, and shift the immune milieu towards autoimmune and cytotoxic responses, or peripheral tolerance [16, 17]. DC are also involved in controlling inherent T cell autoreactivity, contributing to central T cell tolerance [18], and play a critical role in the generation of Treg that suppress effector T cell responses [19, 20]. Human DC migration commences in mid-gestation and fetal immune cells are immunologically-responsive, and may be influenced by trafficked maternal DC in response to intrauterine transplantation [21, 22]. Thus, transient suppression of maternal DC at the time of IUT may enhance chimerism by allowing donor cells to bypass initial antigen recognition and subsequent T cell activation in recipients. We focus on myeloid conventional dendritic cells (cDC, CD11c+ CD123 − ), its subtypes cDC1 (XCR1+), which mediates efficient antigen recognition and cross-presentation to CD8 T cells via MHCI, and cDC2 (CD172a+) which activates CD4 T cells via MHCII, as they are the most frequent DC populations in blood and lymphoid tissues, and influence helper T cell (Th) responses [23–25]. Here, we investigate if long-term engraftment of semi-allogenic and fully allogenic donor cells is influenced by transient maternal DC suppression in a transgenic mouse model of IUT. We interrogated the functional profile, gene expression and T cell and B cell receptor repertoires of trafficked maternal immune cells and the IUT recipient’s immune response to donor cells. We studied this from the perspective of paternal donor cell IUT (pIUT) as this was the most efficient transplantation strategy from our previous study [8], and compared outcomes with maternal donor cell IUT (mIUT) and allogenic donor cell IUT (aIUT).

## Methods

### Animal experiments

Inbred mice strains BALB/c (CD45.2, H2K-d) and C3H/HeNTac (H2K-k referred to as C3H) were obtained from In Vivos (Singapore). C57BL/6 mice (CD45.1, H2K-b, represented as B6) and CD11c-DTR female mice (B6.FVB-1700016L21RikTg^(Itgax−DTR/EGFP)57Lan^/J, CD45.2, H2K-b) were purchased from The Jackson Laboratory (Bar Harbor, ME) and maintained in a specific pathogen-free facility at NUS. BALB/c males and CD11c-DTR females were time-mated for IUT experiments. Pregnant mice were randomly chosen for IUT and downstream experiments. All surgical procedures were conducted under general anaesthesia induced by isoflurane inhalation; mice were euthanised with CO_2_ inhalation for humane endpoints. All animal protocols were approved by Institutional Animal Care and Use Committee (IACUC) at the National University of Singapore (NUS), Singapore (refer to Ethics approval and consent to participate under declarations).

### Transient DC suppression

Non-pregnant CD11c-DTR females were injected with either a single intraperitoneal injection of diphtheria toxin (DT, 5 ng/g of body weight) for transient DC suppression, or saline (control CD11c-DTR). For analysing the immune parameters in these groups, the females were harvested on day 0,1,2,4 and 7 days post injection, and bone marrow (BM), peripheral blood (PB), spleen and uterine horns were analysed for CD4, CD8, CD19, NK1.1, CD11c, MHC-II, XCR1 and CD172a immune parameters by flow cytometry. cDC were identified by the CD11c+ MHC-II+ markers and cDC subpopulation cDC1, cDC2 by XCR1+ and CD172a+ expression respectively.

### Intrauterine transplantation (IUT) and postnatal transplantation

**Fig. 1:**
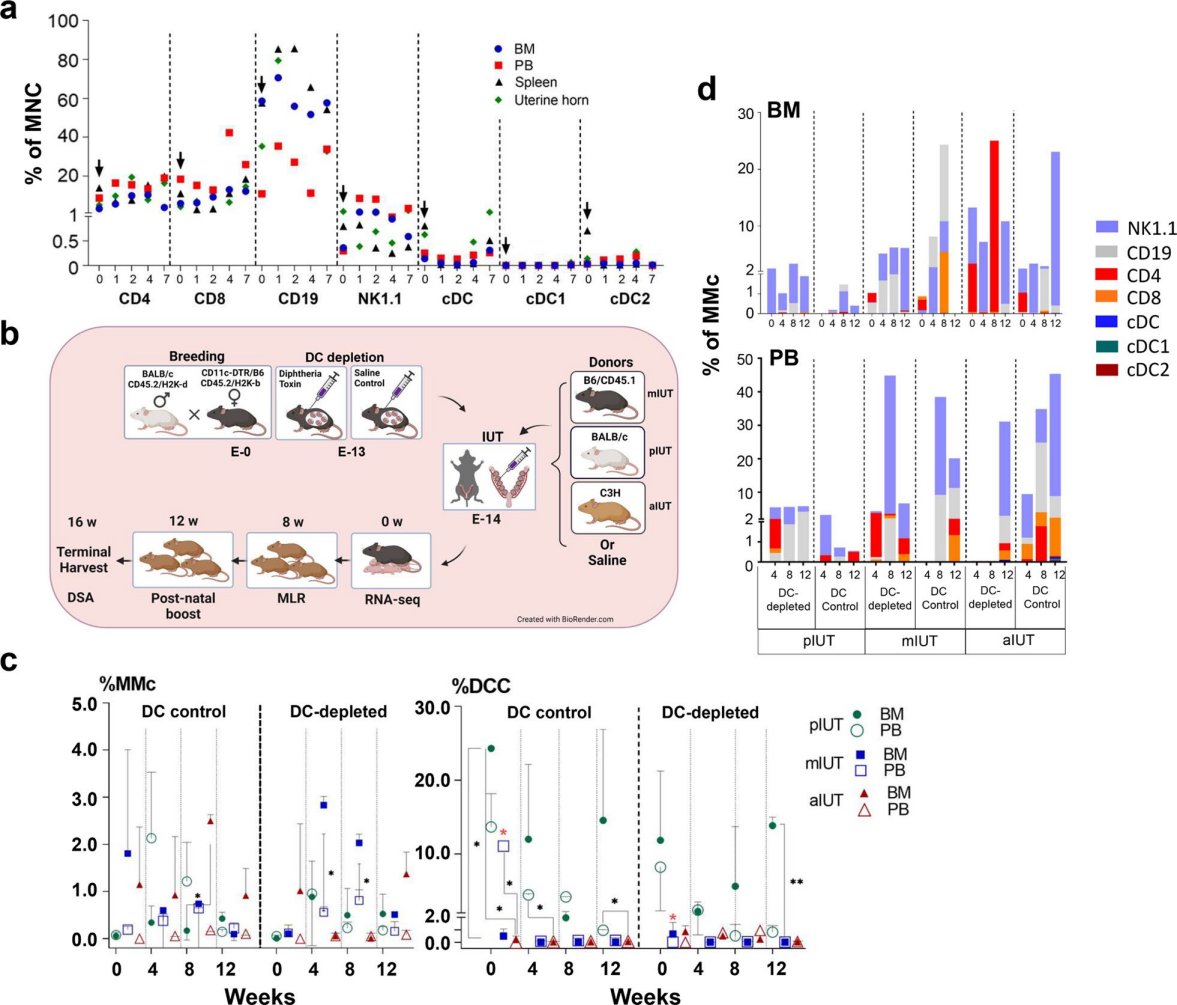
Transient suppression of maternal dendritic cells with diphtheria toxin resulted in reduced maternal immune cell trafficking to fetal recipients following intrauterine transplantation of semi-allogenic and fully allogenic donor cells, and effect on donor cell chimerism. Selective reduction of conventional dendritic cells (cDC) and subtypes (cDC1, cDC2) in various organs of non-pregnant CD11c.DTR females (*n* = 5) after administering diphtheria toxin (DT, arrows, **a**). Schematic diagram representing intrauterine transplantations of maternal (mIUT, *n* = 31) paternal (pIUT, *n* = 60) and fully allogenic (aIUT, *n* = 22) cells performed at E14 (**b**), with or without DC-depletion to the dam. Maternal immune cell microchimerism (MMc) levels were similar in DC-depleted and DC control group with each IUT (**c**), higher in aIUT versus pIUT, and higher in the DC-depleted bone marrow (BM) versus peripheral blood (PB) after mIUT (black*). Donor cell chimerism (DCC) in the DC control group was highest in BM and PB (black*) of pIUT, and higher in mIUT PB vs BM (red*). With the DC-depleted group, DCC was maintained in pIUT BM (black*) and was higher in mIUT BM versus PB (red*). Maternal cDC, CD4, CD19 cells were non-significantly reduced in the DC controls of recipient BM and higher in PB (**d**). cDC and subtypes were only found in both groups of the aIUT recipients. Data represent mean ± SD, analysed by two-way ANOVA with Tukey’s multiple comparisons test

Fresh donor bone marrow mononuclear cells (BM-MNC) were prepared as described previously [8]. The day before IUT (E−13), DT injections (5 ng/g of body weight) were given intraperitoneally to pregnant CD11c-DTR dams to induce transient DC depletion (represented as DC-depleted), while control CD11c-DTR pregnant dams were given saline (DC control). IUT was performed 24 h later (E−14) and all fetuses received an intrahepatic inoculum of 5E+ 6 donor cells, as described previously [8]. Maternal donor cell IUT (mIUT) was performed using B6 BM-MNC (CD45.1, H2K-b), BALB/c BM-MNC was administered in paternal donor cell IUT (pIUT) and C3H BM-MNC were used for completely allogenic donor cell IUT (aIUT). Uninjected fetuses of DC-depleted and DC control groups served as IUT controls (experimental schema shown in Fig. 1b**)**. After littering, pups were nursed by their mothers and weaned at 4 weeks. Fetal cells were harvested from these F1 cross-bred hybrid pups sacrificed between postnatal weeks 1 to 12 to assess DCC, MMc and fetal immunological responses in PB and BM. In selected DC-depleted pIUT offspring, postnatal transplantation (boosting) was performed with 5E+6 donor cells at 12 postnatal weeks administered via the retro-orbital route, without bone marrow ablation or immunosuppression. Serum and organs were harvested 4 weeks later.

### Isolation of Mononuclear Cells (MNC) and Flow Cytometry (FACS) analysis

MNC were isolated from various organs and approximately 1E+6 cells/tube were stained for surface markers to differentiate trafficked maternal cells (H2K-b+), and fetal recipient cells (double positive H2K-b+ , H2K-d+) as reported [8]. Donor cells were identified from fetal recipient cells by the expression of CD45.1+ (maternal donor cells), H2K-d+ (paternal donor cells), H2K-k+ (C3H donor cells) surface markers. We further stained for T cells (expressing CD3, CD4, CD8, NK1.1), B cells (CD19), dendritic cells (CD11c, MHC-II, XCR1, CD172a), HSC markers (Sca1, c-Kit, CD48, CD150), hematopoietic lineage markers (CD11b, GR1, TER119, CD3, CD19, B220). All antibody-stained cells were analyzed using BD X-20 Fortessa™ flow cytometer (BD Biosciences, Franklin Lakes NJ, accessed at the NUS Life Science Institute), The list of antibodies used for flow cytometry are provided in Additional file 1: Table (S1). Each antibody was validated with positive control samples as per manufacturer’s instructions. All raw data files were compensated and analysed using FlowJo™ software (FlowJo LLC, Ashland OR). Percentages of donor cells and MMc were calculated from total live MNC. Percentages of immune populations were calculated from the respective total population of MMc, donor cells and recipient cells, and percentages of HSC markers were calculated from total donor cells.

### Mixed lymphocyte reactivity assay (MLR) for functional T cell activity

Splenocytes (responder MNCs) were isolated from 4 to 6 week old F1 cross-bred IUT (from both DC control and DC-depleted groups) and naive pups (from uninterrupted pregnancies). Lymphoid tissue MNC from BALB/c, B6 or C3H mice, treated with mitomycin C (50 µg/ml), served as stimulator cells. MLR was performed as reported [8]. Cells were harvested 72 h post-culture and analysed for T cell subset surface markers (CD4, CD8, CD25, CD62L, CD44) and intracellular marker FOXP3 in the responder cells by flow cytometry. Markers for phenotypes were: central memory T cells (central Tm, CD44+ CD62L+), effector memory T cells (effector Tm, CD44+), effector T cells (Teff, CD25+) and regulatory T cells (Treg, FOXP3+). A part of the cells was used for RNA extraction and subsequently analyzed for cytokines and FOXP3 gene expression by RT-qPCR.

### Quantitative gene expression analysis

Total RNA extraction and qPCR was performed as described previously [8]. The qPCR primers used are provided in Additional file 1: Table S2.

### Bulk RNA sequencing

Trafficked maternal and recipient cells were isolated from whole-body harvests (including primary and secondary lymphoid organs) of 1-week-old F1 hybrid neonates, by magnetic cell sorting (MACS), followed by FACS, using H2K-b and H2K-d antibodies respectively. Due to the limited number of trafficked maternal cells harvested from each neonate, samples were pooled together for RNA-seq analysis and thus there are no replicates for this analysis. Total RNA was isolated from sorted cells using RNeasy micro kits (Qiagen, Hilden, Germany) with on-column RNase-free DNase digestion and eluted carrier-free. Total RNA was quantified, and quality assessed using Agilent 2100 Bioanalyzer (Agilent Technologies Inc., Santa Clara, CA), before cDNA library preparation. Samples were sequenced on a flow cell using HiSeq 4000 system (Illumina, San Diego, CA), with a read length of 150 bp and 100 million reads per sample. The raw reads were analysed with the RNAseq pipeline from nf-core [26] using the reference genome (GRCm39) and gene annotation (M26) from GENCODE (https://www.gencodegenes.org/). The gene read count table generated by featureCount in the RNAseq pipeline was analysed using edgeR software [27, 28]. The dispersion value was set at 0.4 as recommended in edgeR for experimental setups with no replicates.

### Gene expression analyses

Using log of fold-change (logFC) data, we selected the most up- and down-regulated genes (1500 each, for a total of 3000) from each treatment group to organize using an online Venn diagram generator (http://bioinformatics.psb.ugent.be/webtools/Venn/) to single out common genes across all the groups. We analysed functions of enriched genes using the Database for Annotation, Visualisation and Integrated Discovery (DAVID v6.8, https://david.ncifcrf.gov/) [29, 30].

### TCR and BCR repertoire analyses

Raw reads were analyzed by MIXCR performed with settings of analysis of random fragments, RNA starting material and using the provided Mus musculus dataset, all others remaining as default settings [31, 32]. Results from MIXCR were imported into VDJtools (https://github.com/mikessh/vdjtools) to plot the clonotypes in PlotQuantileStats [33].

### Detection of donor-specific antibodies (DSA)

Serum collected from harvested pups and serum collected 4 weeks after postnatal transplantation were used for DSA. For generating positive control sera, wild-type B6 mice were sensitized with maternal (B6/CD45.1), paternal (BALB/c) or allogenic (C3H) splenocytes at a dose of 2E+ 7 cells, injected intra-peritoneally at an interval of 2 weeks. Sera was collected after 14 days. DSA assay was performed using the respective splenocytes, as described previously [8].

### Statistical analyses

**Fig. 2:**
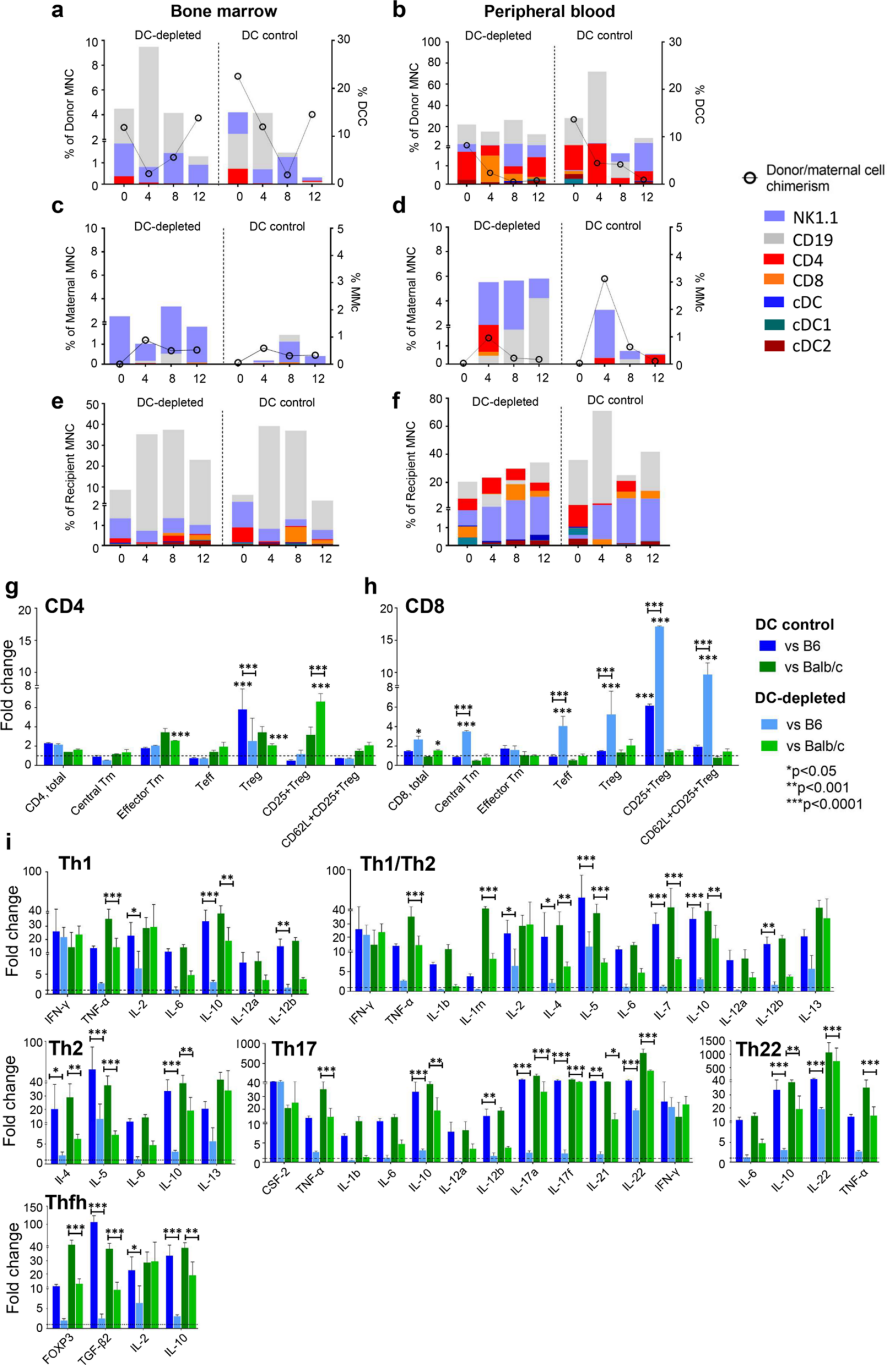
Immune profiles of engrafted donor, trafficked maternal and recipient cells following paternal cell transplantation and immune T cell responses, cytokine and FOXP3 gene expression following re-exposure to donor cells. Compared to DC control (*n* = 9), DC-depleted recipients (*n* = 11) had engrafted donor cells showing reductions in all immune types relative to CD19 (p < 0.05, **a**, **b**), and no significant changes in the trafficked maternal immune cells in bone marrow (BM) and peripheral blood (PB) (**c**, **d**). Recipient immune cell profile was similar in DC control and DC-depleted groups, with relatively higher CD19 in both (*p* < 0.005, **e**, **f**). Postnatal weeks are shown on the x-axis (**a**–**f**). DC-depleted pups (*n* = 4) showed higher fold-changes in CD4+ effector memory (Tm), regulatory T cells (Treg) and CD25+ Treg compared to uninjected controls (*n* = 3, represented by the dotted horizontal line) and higher CD25+ Treg compared to DC control (*n* = 4), when exposed to BALB/c donor cells (**g**). Higher fold changes in CD8 central memory (Tm), effector (Teff), Treg and CD62L+ CD25+ Treg were observed in DC-depleted pIUT pups compared to DC control and uninjected control (*n* = 4) when stimulated with B6 cells (**h**). Reductions in helper T cell cytokines and FOXP3 levels **i** were observed with DC-depletion (*n* = 4) in response to paternal and maternal donor cells, compared to DC control (*n* = 4). The horizontal dotted line represents levels in the uninjected control group normalised to a value of 1 (**g**–**i**). Bars with dark shades represent DC control pups and light shades represent DC-depleted pups (**g**–**i**). Data represent mean ± SD, analysed by two-way ANOVA with Tukey’s multiple comparisons test

**Fig. 3:**
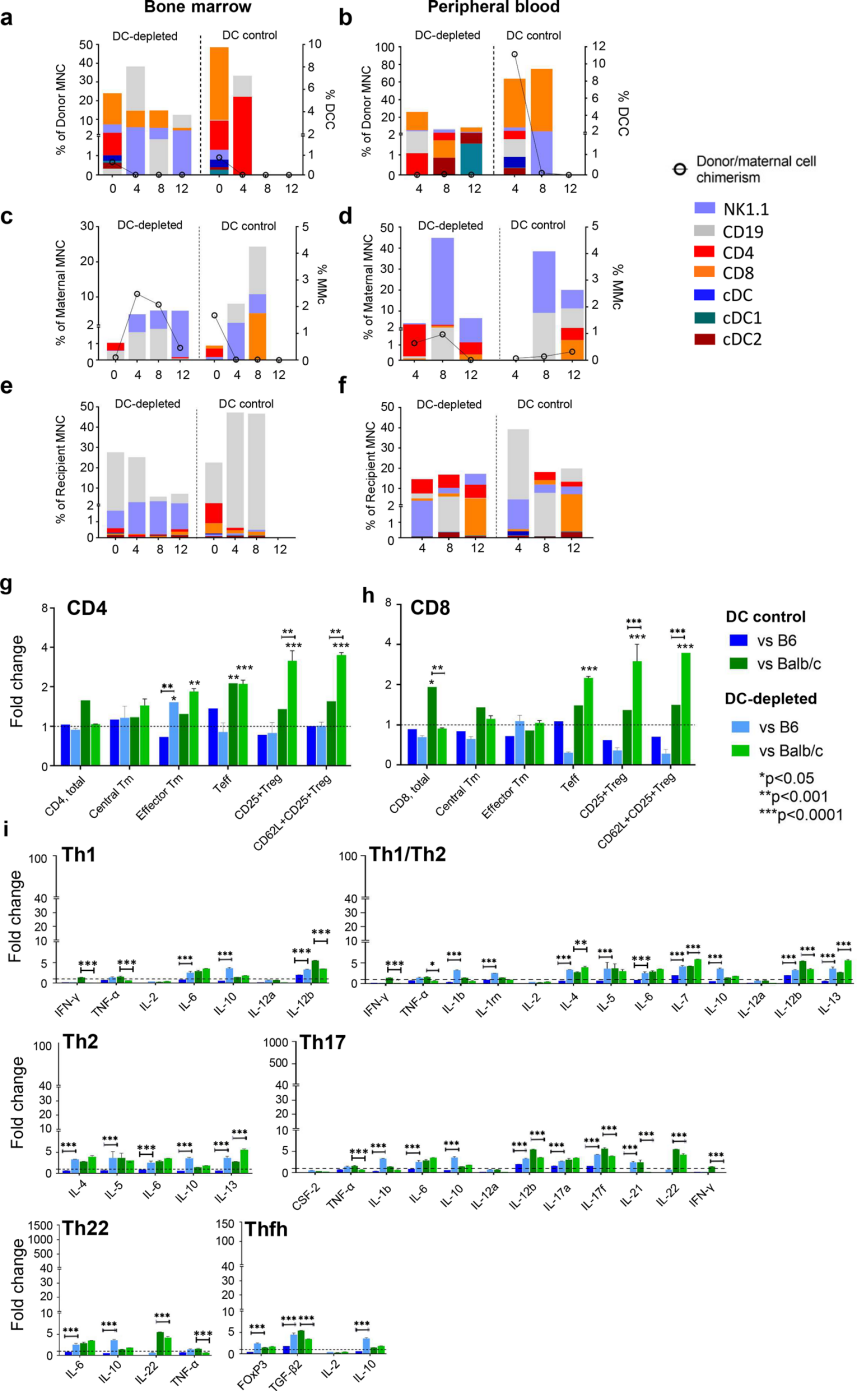
Immune profiles of engrafted donor, trafficked maternal and recipient cells following maternal cell transplantation and immune T responses, cytokine and FOXP3 gene expression following re-exposure to donor cells. Donor cell chimerism was low in DC-depleted (*n* = 8) and DC control groups (*n* = 7). In DC-depleted, CD8, CD19 were higher in BM and CD8 lower in PB (*p* < 0.05) than in DC control group (**a**, **b**). MMc (**c**, **d**) and recipient immune cell profiles (**e**,** f**) were similar in both DC groups. Postnatal weeks are shown on the x-axis (**a**–**f**). Within DC-depleted group (*n* = 4), CD4+ effector Tm, CD4+ and CD8+ effector (Teff) were increased when compared to uninjected control, while CD4 and CD8 regulatory T cells were increased when compared to DC control pups (*n* = 4) and uninjected controls (*n* = 3) following paternal donor cell exposure (**g**, **h**). DC-depleted (*n* = 4) and DC control (*n* = 4) produced similar low-level responses in individual cytokines when compared to uninjected controls (*n* = 2) which were much lower than in paternal IUT (see Fig. 2i for comparison). The DC-depleted group produced higher expression of largely inhibitory cytokines associated with Th2, Th1/Th2, Th17 cells, following B6 stimulation (**i**). The horizontal dotted line represents levels in the uninjected control group normalised to a value of 1 (**g**–**i**). Bars with dark shades represent DC control and light shades represents DC-depleted pups (**g**–**i**). Data represent mean ± SD, analysed by two-way ANOVA with Tukey’s multiple comparisons test

**Fig. 4:**
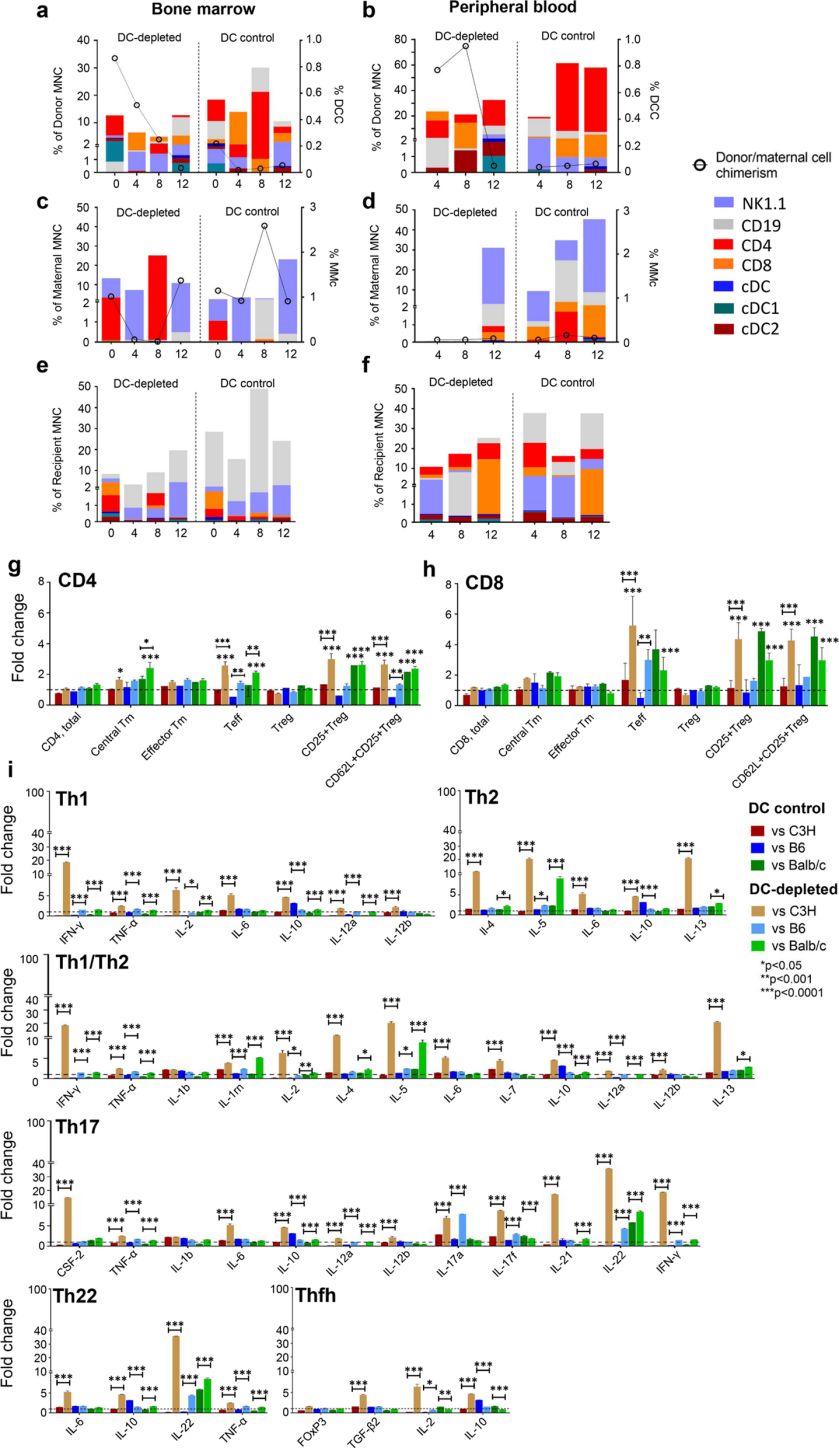
Immune profiles of engrafted donor, trafficked maternal and recipient cells following allogenic (C3H) donor cell transplantation and immune T cell responses, cytokine and FOXP3 gene expression following re-exposure to donor cells. Donor microchimerism < 1% was observed in BM and PB (**a**, **b**). No differences in donor, maternal (**c**, **d**) or recipient (**e**, **f**) immune cell profiles were observed in DC-depleted and DC control groups. Within the DC-depleted group, CD4+ central memory (Tm), CD4+ and CD8+ effector (Teff) and regulatory T cells CD25+ Treg and CD62L+ CD25+ Treg were elevated above the DC control group and uninjected controls on exposure to C3H, B6 or BALB/c donor cells (**g**, **h**). Cytokine levels from DC-depleted aIUT recipients were significantly higher compared to DC control pups when stimulated with C3H, B6 or BALB/c donor cells, except for IL-1b and FOXP3 (**i**). The horizontal dotted line represents levels in the uninjected control group normalised to a value of 1 (**g**–**i**). Data represent mean ± SD, analysed by two-way ANOVA with Tukey’s multiple comparisons test

Continuous data were analysed using Analysis of variance (2way ANOVA) with Tukey’s multiple comparisons test, with a single pooled variance and multiple *t* tests for comparisons of individual parameters. Statistical significance was determined at *α* = 5.0%. Values are expressed as mean ± standard deviation (SD). Pearson correlation coefficient was also used assuming linear relationships between the variables tested. Analyses were performed with GraphPad Prism version 9 for Windows (GraphPad Software, San Diego, CA, www.graphpad.com). The datasets generated and/or analysed during the current study are available from the corresponding author on reasonable request. Error bars were not provided on Figs. 2a–f, 3a–f and  4a–f, as they may mask a clear view of the graph bars. Instead, respective raw data (mean ± SD), were provided in Additional file 1: Tables S3–5).

## Results

### Maternal CD11c+ MHC-II+ DC depletion influenced trafficked maternal cells following IUT.

DT administration to non-pregnant CD11c-DTR females depleted DC in spleen, BM, PB and uterus (flow cytometry plots showed on Additional file 1: Figure S1a) [34]. Baseline cDC (0.23–0.38%) reached troughs (0.01–0.06%) 2–7 days post-DT injection, returning to baseline on day 7 in BM and uterus (Fig. 1a). cDC1 was < 0.1% and cDC2 was < 0.5% at all time points in BM. Other immune cells remained unaffected by DT, except CD19 levels which were transiently elevated for 24–72 h post-administration, returning to baseline by day 7. Our IUT model (CD11c-DTR females crossed with BALB/c males) allowed us to track donor, maternal and recipient cells separately by flow cytometry (Additional file 1: Figure S1b). The survival rates of experimental pups in DC control and DC-depleted groups respectively were 92.0% and 76.9% (non-IUT controls, *n* = 61), 75.0% and 79.6% (pIUT, *n* = 60), 81.1% and 45.0% (mIUT, *n* = 31), 100.0% and 46.7% (aIUT, *n* = 22).

Overall, MMc in both DC-depleted and DC control pIUT were similar in recipient BM (0.32–0.48%) and PB (0.43–0.78%). MMc was higher in BM than PB in DC-depleted mIUT (at 4-8w, *p* < 0.05, Fig. 1c), and MMc in BM was higher in aIUT than pIUT (DC control, at 8w, *p* < 0.05, Fig. 1c and d). cDC was observed only in the BM of DC control aIUT (0.03–0.08%), and in the PB of DC-depleted and DC control aIUT (0.04–0.08%). cDC1 and cDC2 were detected only in BM of DC control mIUT and aIUT (0.03–0.04%, Fig. 1d). Compared to DC controls, we observed non-significant reductions in CD4, CD8 and CD19 in the BM of DC-depleted pIUT and DC-depleted mIUT, but there was an increase in CD4 and CD8 in the BM of DC-depleted aIUT (Fig. 1d). There were non-significant increases in CD4 and CD8 in the PB of DC-depleted pIUT and DC-depleted mIUT. However, there were reductions in all cells in DC-depleted aIUT, particularly NK1.1 (10.4 ± 10.4% vs. 29.9 ± 10.7%, *p* < 0.0001). Thus, the correlation of MMc and maternal DC depletion appears to be related to the donor IUT.

### Donor cell chimerism was highest following pIUT and hematopoietic markers were retained

Among DC control recipients, we observed higher DCC in BM (DCC^BM^) in pIUT than in mIUT and aIUT recipients (0w, *p* < 0.05). We observed higher DCC in PB (DCC^PB^) in DC control pIUT compared to aIUT (4–12w, *p* < 0.05), and higher DCC^PB^ following mIUT when compared with DC-depleted mIUT and aIUT (0w, *p* < 0.05). With DC-depleted pIUT, DCC^BM^ was maintained > 2% until 12w, though DCC^PB^ declined to < 1% by 8w (DCC^BM^ v DCC^PB^ at 12w, *p* < 0.001, Fig. 1c). DCC^BM^ in both DC control and DC-depleted mIUT remained < 1%, while DCC^PB^ declined rapidly from 11.07 ± 0.07% at 0w onwards in DC control mIUT, and remained < 1% from 0 to 12w in DC-depleted mIUT. DCC^BM^ and DCC^PB^ in both DC control and DC-depleted aIUT remained < 1% (0–12w). Recovered chimeric donor cells in BM and PB from DC control and DC-depleted pIUT recipients showed similar proportions of LSK cells, indicating the presence of long-term repopulating HSC. LSK cells in DC control and DC-depleted BM-engrafted cells comprised 1.1% and 3.9% of total cells, LSK CD48+ (haemopoietic progenitors) comprised 0.5% and 1.1%, LSK CD150+ (long-term repopulation) comprised 0.1% in both, and LSK CD48+ CD150+ ranged from 0 to 0.6%, respectively. In DC control and DC-depleted PB-engrafted cells, LSK made up ~ 0.5% of the population in both groups, LSK CD48+ ranged from 0.01 to 0.08% respectively, LSK CD150+ comprised 0.3% and LSK CD48+ CD150+ comprised 0.01% in both groups. In recipient bone marrow cells from mIUT (week-0) and pIUT (week-4) pups, expression of LSK CD48+ , CD48+ CD150+ and CD150+ were similar, as were the lineage markers (CD11b, GR1, TER119, CD3), among DC control and DC-depleted groups (Additional file 1: Figure S1c, d). We deduced that post-IUT maternal cell trafficking was an active process, as we observed significant reductions in CD3 and CD19 in recipient BM and PB compared to uninjected dams (all differences, p < 0.0001), except for NK1.1 which was higher in aIUT recipients than controls (*p* < 0.0001, Additional file 1: Figure S2a).

### Maternal DC depletion increased recipient Treg and reduced cytokine expression associated with alloreactivity

We next examined the impact of maternal DC depletion on DCC and alloresponsiveness of IUT recipients. In DC-depleted pIUT, CD19 was significantly higher in DCC^BM^ (4–8w, *p* < 0.05) and in DCC^PB^ (0–12w, *p* < 0.05) than other cells. In DC control IUT recipients, CD19 in DCC^PB^ was more prevalent at 0w (*p* < 0.0001, Fig. 2a, b). Within corresponding MMc, maternal CD3 was significantly lower in DC-depleted pIUT compared to DC control pIUT (where it is the most prevalent cell) at 0–12w (*p* < 0.005), and CD3 was also higher than cDC subsets in DC-depleted pIUT (4w, *p* < 0.05, data not shown); no other differences were observed in MMc between groups (Fig. 2c, d). DC-depleted and DC control pIUT recipients produced higher CD19 than other immune cells in BM and PB (*p* < 0.005 at all-time points, Fig. 2e, f) with no differences between groups. In vitro assessment of functional tolerance was performed by MLR at 8w when DC-depleted pIUT recipients had waning DCC^PB^ at < 1%, though DCC^BM^ was maintained. Here, re-exposure of DC-depleted pIUT splenocytes to BALB/c cells elicited greater expression of CD4 effector memory (Tm), regulatory (Treg) and CD25+ Treg compared to uninjected controls (dotted horizontal line, *p* < 0.001, Fig. 2g). Re-exposure to B6 cells on the other hand did not produce significantly different CD4 readouts compared to uninjected controls, though CD4 Treg were significantly lower in DC-depleted compared to DC control groups (*p* < 0.001). DC-depleted pIUT recipients showed greater fold-change in CD8 central memory Tm, effector (Teff), Treg and CD62L+ CD25+ Treg (*p* < 0.001) when challenged with B6 cells, compared to DC control pIUT and uninjected fetuses (Fig. 2h). DC control pIUT produced greater fold-changes in cytokines associated with helper T-cells [35] when stimulated with BALB/c or B6 cells, while a relatively lower response was observed for DC-depleted pIUT (Fig. 2i**)**.

In mIUT, although donor cells from maternal BM-MNC and trafficking maternal cells are both considered autologous, DC within the injected BM-MNC is immature, and cannot elicit proinflammatory immune responses in the fetal microenvironment compared to circulating mature DC trafficking from maternal peripheral blood [36]. Therefore, we do believe there is some merit to transiently depleting maternal DC, even in the mIUT experimental group. Though DCC^BM^ and DCC^PB^ were < 0.2% in DC-depleted mIUT (Fig. 3a, b), we were able to analyse donor immune cell components. DCC^BM^ in DC-depleted mIUT contained higher CD8 and CD19 than cDC and NK1.1 (*p* < 0.005. Figure 3a); compared to DC control mIUT, CD4 was lower and NK1.1 was higher in DC-depleted bone marrow (*p* < 0.005), with no differences between other cell types. There were no differences in proportions of any cell type in DCC^PB^ following DC-depleted mIUT; compared with DC control mIUT, CD8 was lower in DC-depleted peripheral blood (*p* < 0.0001, Fig. 3b). There were no differences in MMc (Fig. 3c, d) and recipient immune profile (Fig. 3e, f) in the BM or PB of DC-depleted mIUT compared to DC control mIUT.

DC-depleted mIUT produced elevated CD4 effector Tm, CD4 and CD8 Teff, and CD4 and CD8 CD25+ Treg, CD62L+ CD25+ Treg when stimulated with BALB/c cells only on MLR (Fig. 3g, h); only low-level responses to B6 cells were elicited, except for higher CD4 effector Tm compared to uninjected and DC control groups. Cytokine expression was much lower in DC control mIUT than pIUT recipients (< sixfold increase in mIUT (Fig. 3i) vs. > twofold increase in pIUT (Fig. 2i) over uninjected controls). DC-depleted mIUT produced a higher expression of largely inhibitory proteins (IL5, IL6, IL10, FOXP3, TGFβ2) in combinations associated with Th2, Th1/Th2, Th17 cells, following B6 stimulation (Fig. 3i).

In contrast, DC-depleted and DC control aIUT resulted in only microchimeric DCC^BM^ and DCC^PB^, with similar donor immune cell profiles (Fig. 4a, b). No differences in MMc, trafficked maternal immune cell profile or recipient immune cell profile were observed (Fig. 4a–f). Significant fold-change increases in CD4 central memory Tm, CD4 and CD8 Teff, CD25+ Treg, and CD62L+ CD25+ Treg were observed in DC-depleted aIUT on MLR (Fig. 4g, h), which also significantly increased expression of cytokines and regulatory proteins except FOXP3 when stimulated with C3H, compared to DC control aIUT, implying proimmune helper T cell enhancement.

Except for IL-1b and FOXP3, cytokine levels from DC-depleted aIUT recipients were significantly higher compared to DC control pups when stimulated with C3H, B6 or BALB/c donor cells (Fig. 4i). We attempted to evaluate regulatory B-cells (Breg) which lack unique phenotypic markers [37, 38]. pIUT and mIUT with and without DC depletion augmented IL10, IL5, IL6, TGF-β2, FOXP3 expression (Figs. 2i and  3i) suggesting the presence of activated Breg in response to IUT. There were no differences in CD19 post-transplantation (Figs. 2e, f and  3e, f).

### IUT and DC depletion influence maternal and recipient T- and B-cell receptor repertoire diversity

**Fig. 5:**
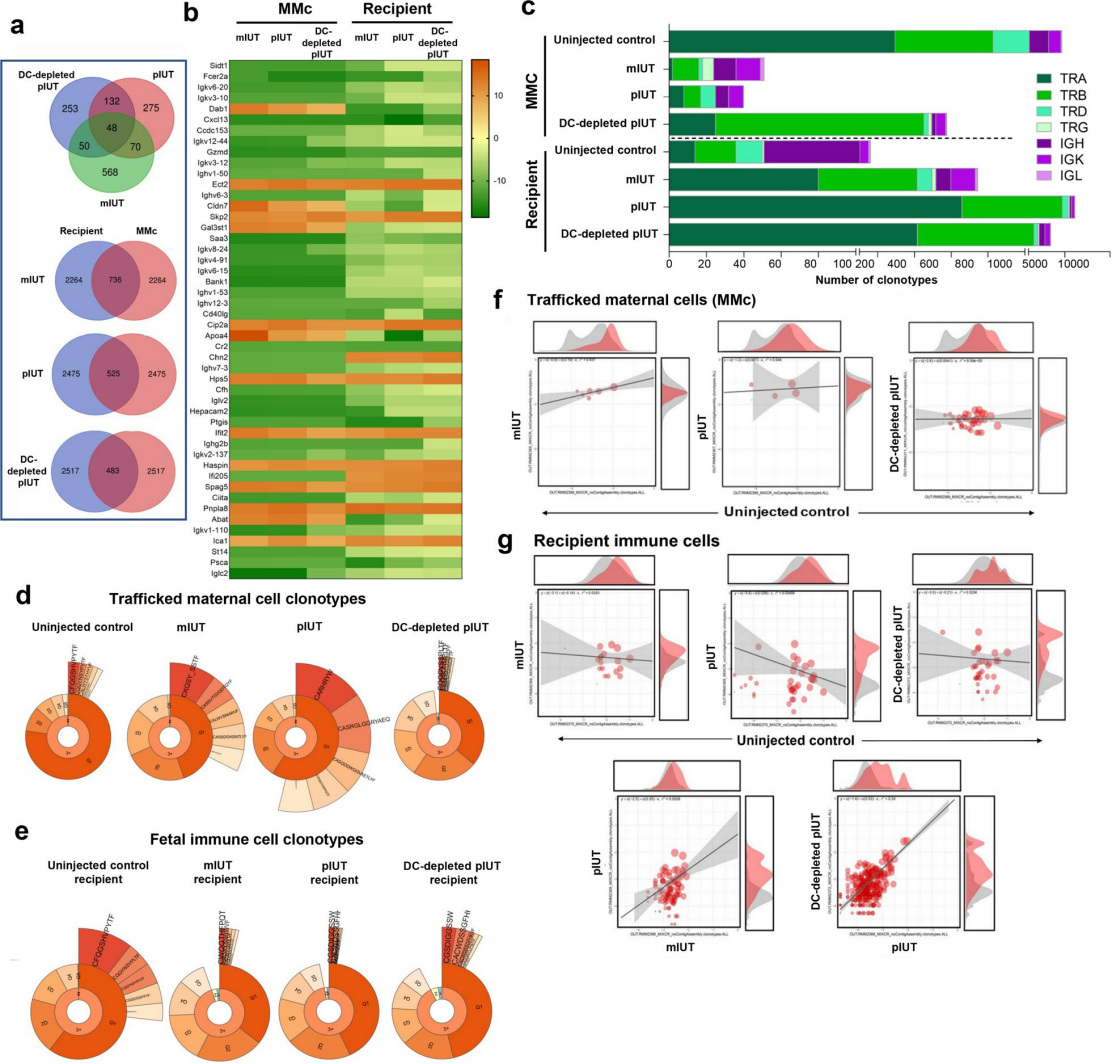
Gene expression profile and T cell (TCR) and B cell (BCR) receptor repertoire of trafficked maternal and recipient immune cells following IUT. Trafficked maternal and recipient immune cells from pIUT (DC control, *n* = 5), DC-depleted pIUT (*n* = 5), mIUT (DC control, *n* = 7) shared 48 genes (top panel) and up to 30% genes (bottom panel) within each IUT (**a**). Common gene clusters represent cell adhesion, cytokine response and immunoregulatory pathways (**b**). Trafficked maternal and recipient-derived BCR and TCR clonotypes (**c**) were similar in uninjected pups (*n* = 17); IUT increased TRB, TRG, reduced TRA, TRD, IGH, IGK in maternal-derived and recipient-derived clonotypes. Top 5 maternal-derived clonotypes were greatest following IUT and contracted in DC-depleted pIUT (**d**). Recipient-derived top 5 clonotypes were most abundant in uninjected pups (**e**). Large overlaps observed between DC-depleted pIUT and uninjected maternal-derived clonotypes (**f**) and between IUT recipients (**g**)

To further parse maternal and recipient immune interactions, we analysed gene expression profile and T-cell (TCR) and B-cell (BCR) receptor repertoires in MNC isolated from pIUT and mIUT recipients. Cells harvested from animals within a group were pooled for analyses given the limited quantity of cells obtained. Pooled cells represent the following numbers of animals: uninjected offspring (no IUT, *n* = 17), DC control mIUT (*n* = 7), DC control pIUT (*n* = 5), and DC-depleted pIUT (*n* = 5). We had insufficient cell harvests from DC-depleted mIUT, DC-depleted and DC control aIUT, precluding analyses. Top 3000 up- and down-regulated genes in each group were identified by enrichment score (ES) > 1.3. Within treatment groups, recipient and maternal cells shared 483–736 common genes (Fig. 5a). With DC control mIUT, 26/174 enriched recipient gene clusters and 25/171 maternal clusters represented RNA and protein metabolism, hemopoiesis and immune system development, also represented by 21/169 recipient clusters and 49/177 maternal clusters following DC control pIUT. With DC-depleted pIUT, 20/153 recipient clusters additionally represented T-cell regulation. Upregulated DC control pIUT and DC-depleted pIUT maternal clusters represented mitogen-activated protein kinases (MAPK) cascades, T-cell activation, and immune system development. All groups shared 48 common genes, of which 6/40 highly enriched clusters represented cytokine stimulus response, immune system regulation, B-cell mediated immunity and adaptive immune response (Fig. 5b).

Compared to uninjected controls, retrieval frequency of maternal-derived TCR and BCR clonotypes increased from 0.02 to 2.04–3.13% following mIUT and pIUT respectively (Table 1), and these showed reduced diversity, with Hill numbers (order 1, exponential of Shannon–Wiener indices)[39] of 18–29, compared to 941 in controls. DC-depleted pIUT decreased maternal clonotype retrieval by 17.3-fold and restored diversity towards baseline (Hill number of 447). In contrast, DC control mIUT and pIUT recipient-derived clonotypes (retrieval frequency 0.02–0.15%) showed increased diversity (Hill numbers 528–3728 vs. 64 in uninjected controls). DC-depleted pIUT reduced diversity in recipients (Hill numbers from 3728 to 1637).

**Table 1:** RNAseq analysis of trafficked maternal and recipient cells for TCR and BCR repertoire clonotypes

Sample	Total counts	Clonotype number	Mean clonotype frequency	Hill numbers (order 1) exponential of the Shannon-Weiner Index
*Trafficked maternal immune cells*				
Uninjected pup	348,684	4429	2.26E−4	941
DC control mIUT	8376	49	2.04E−2	29
DC control pIUT	3749	32	3.13E−2	18
DC-depleted-pIUT	5537	552	1.81E−3	447
*Fetal immune cells*				
Uninjected pup	66,848	148	6.76E−4	64
DC control mIUT recipient	4043	666	1.50E−3	528
DC control pIUT recipient	48,175	5488	1.82E−4	3728
DC-depleted-pIUT recipient	18,579	3064	3.26E−4	1637

Maternal- and recipient-derived TCR and BCR clonotypes were similar in uninjected pups; BCR immunoglobulin heavy (IGH), κ (IGK), *λ* (IGL) chains made up ~ 81% of maternal and 75% of recipient clonotypes (Fig. 5c). Expansion of TCR β-chain (TRB) clonotypes and reduction in IGH were observed among maternal-derived clonotypes following mIUT and pIUT. DC-depleted pIUT produced further increases in TRB, TRA (α-chain), TRD (δ-chain), IGH, IGK relative to DC control pIUT. Among recipient-derived clonotypes, we observed expanded TRB, TRG (γ-chain), and reduced IGH, IGK following DC control mIUT and pIUT, and DC-depleted pIUT further reduced TRB and increased IGK and IGH.

Mostly higher order (3+) clonotypes were encountered with IUT (Fig. 5d, e). Top 20% of clonotypes (Quantile 1, Q1) were most abundant in uninjected controls, and clonotypes in Q2 to Q5 were more abundant in IUT recipients. Individual abundances of top 5 maternal-derived clonotypes in each group were expanded with IUT and diminished with DC depletion (Fig. 5d), while recipient-derived top 5 clonotypes were most abundant in uninjected pups (Fig. 5e). We observed a large number of public maternal-derived clonotypes between DC-depleted pIUT and uninjected controls (Fig. 5f), and a substantial number of public recipient-derived clonotypes between DC control mIUT and pIUT, and DC-depleted pIUT (Fig. 5g).

**Fig. 6:**
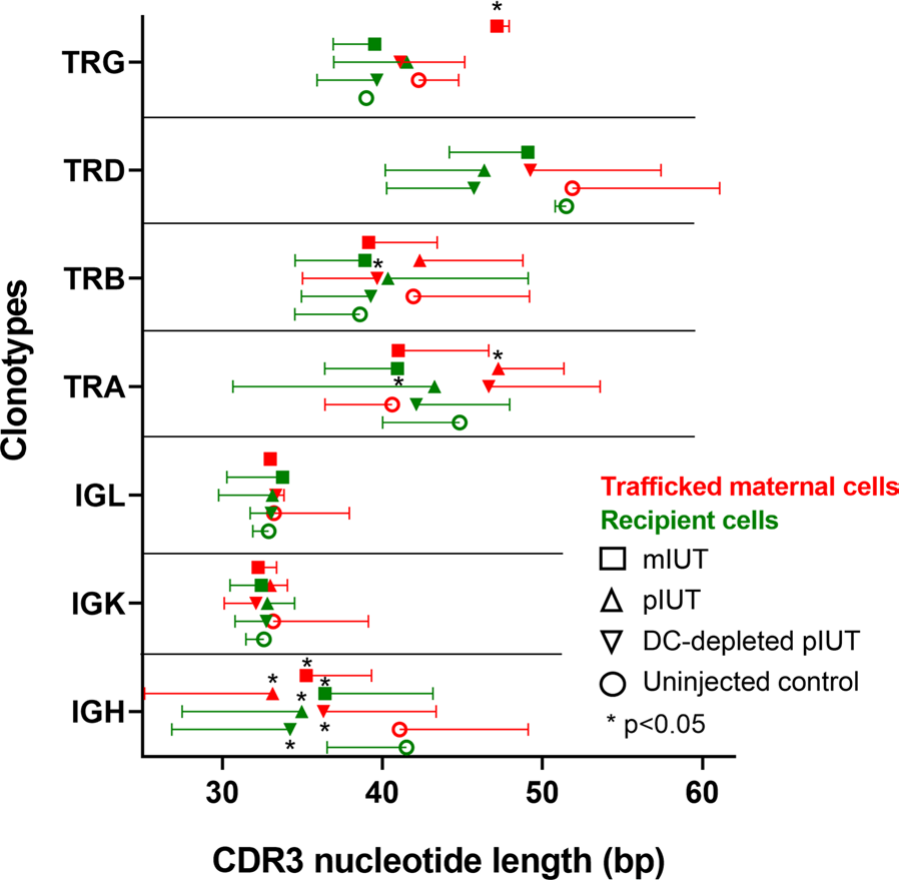
TCR and BCR clonotypes show CDR3 nucleotide length changes in response to IUT. Increased CDR3 nucleotide sequence lengths were observed in maternal TRG (from mIUT), TRA (pIUT, DC-depleted pIUT), and shorter lengths in maternal TRB (DC-depleted pIUT), recipient TRA (mIUT), and maternal and recipient IGH (any IUT), compared to uninjected control pups. Mean ± SD are compared against controls, analysed with paired *t*-test. bp: base pairs

**Fig. 7:**
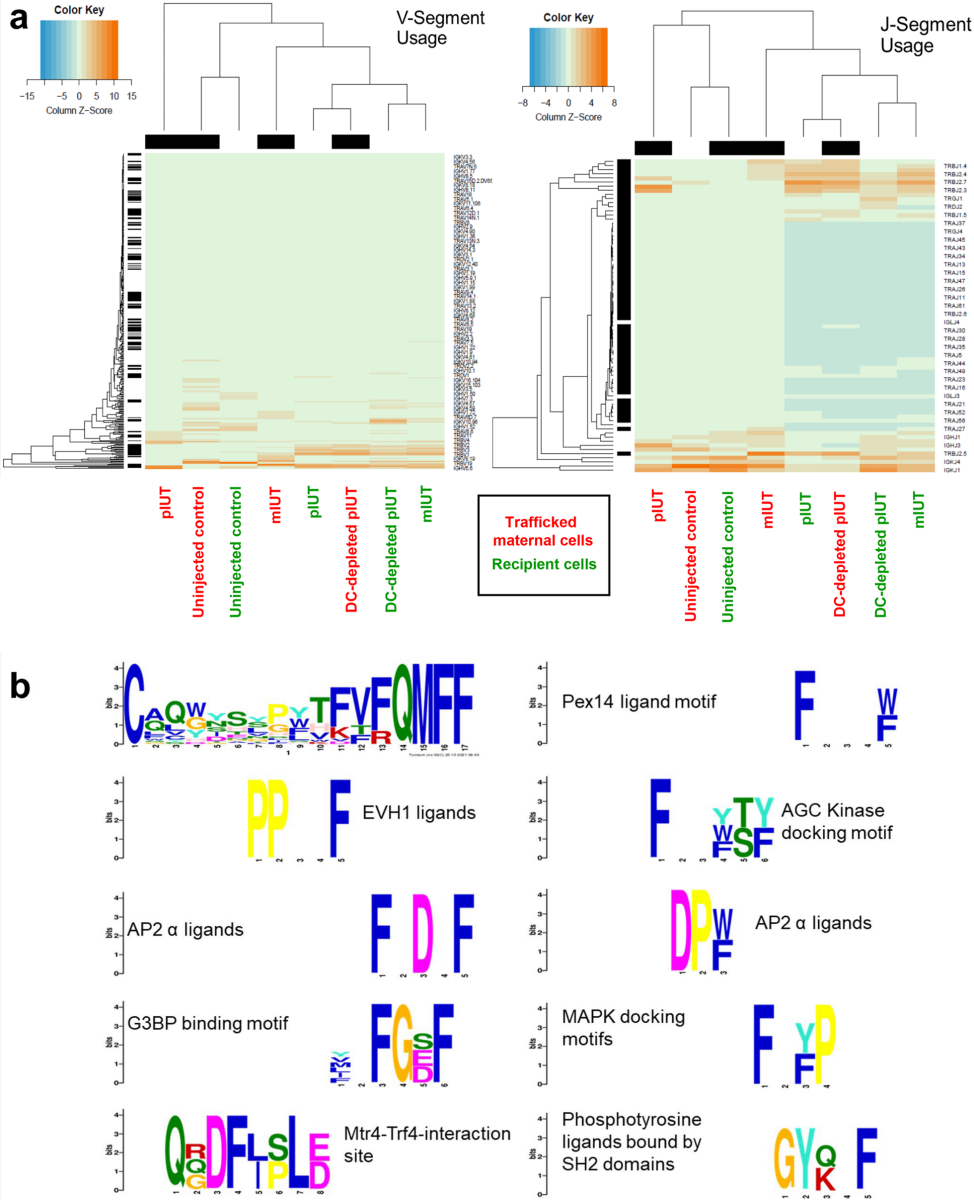
CDR3 V-segment and J-segment analyses and functionality in the trafficked maternal (MMc) and recipients of IUT groups. Similarities in variable V-segment and joining J-segment usage were observed between pIUT, DC-depleted pIUT and mIUT recipient-derived clonotypes and DC-depleted pIUT MMc-derived clonotypes, with upregulated IGH, IGK, TRA, TRB V- and J-segments, and TRG and TRD J-segments; TRA J-segments were downregulated (**a**). CDR3 amino acid sequences of the top 20 shared clonotypes demonstrated motif enrichment (listed in **b**) corresponding to Ras GTPase activating proteins, mitogen-activated protein kinases (MAPK) substrates, and signal transduction molecules, among others

Complementarity-determining region 3 (CDR3) incorporates the VDJ recombination junctions, accounting for most of the repertoire variation mediating specific antigen recognition [40]. CDR3 regions of naïve and antigen-experienced clonotypes are longer and shorter respectively, reflecting antigen-driven selection [41]. We found a significant shift towards longer CDR3 in maternal TRG (from DC control mIUT) and TRA (DC control pIUT, DC-depleted pIUT), and shorter CDR3 in maternal TRB (DC-depleted pIUT), recipient TRA (DC control mIUT), and maternal and recipient IGH (all groups, Fig. 6**)**. Analyses of gene segment usage to functionally characterize these clonotypes by hierarchical clustering indicate similarities in V/J-segment usage between DC control pIUT, DC-depleted pIUT and DC control mIUT recipient-derived clonotypes, and DC-depleted pIUT maternal-derived clonotypes, where IGH, IGK, TRA, TRB V/J-segments, and TRG and TRD J-segments, were upregulated and TRA J-segments were downregulated. We also observed uniqueness in V/J-segment usage within uninjected controls, DC control mIUT and all pIUT clonotypes (Fig. 7a). Functionality of the top 20 shared clonotypes, assessed by comparing CDR3 amino acid sequences to protein databases using Tomtom [42], revealed motif enrichment corresponding to production of Ras GTPase activating proteins, MAPK substrates, and signal transduction molecules, among others (Fig. 7b).

### Postnatal transplantation following maternal DC depletion and IUT demonstrated sustained donor-specific hyporesponsiveness at 16w

DC-depleted pIUT recipients were postnatally transplanted with either parental donor cell without immunosuppression to assess their functional tolerance to these cells in vivo. At 12w postnatal, these offspring had microchimeric DCC^PB^ but maintained DCC^BM^ > 10.0% (Fig. 2a–d). Postnatal challenge of B6 maternal cells at 12w increased recipient CD3 and decreased CD19 in lymph nodes, relative to challenges with BALB/C paternal cells and saline only (Additional file 1: Figure S2b–f). No differences in other immune cells and no donor-specific IgG or IgM antibodies were observed at 16w postnatal (4 weeks post-transplantation, Additional file 1: Figure S2g).

## Discussion

To our knowledge, this is the first report of maternal immune cells “primed” on exposure to donor cells influencing recipient immune responses to IUT. Our current data support our previous observations that paternal donor cells engraft more efficiently, and that donor-specific tolerance is acquired from IUT of donor BM from either parent. Though maternal DC depletion prior to IUT does not affect donor chimerism, we further show that trafficked maternal immune cells influence host immune response to IUT. Actively trafficked maternal cells demonstrate reduced TCR and BCR diversity, suggesting the expansion of specific clonotypes following exposure to parental donor cells, while maternal DC depletion restored diversity, particularly of maternal T clonotypes. While recipient clonotype diversity was preserved with maternal DC depletion, offspring showed enhanced tolerogenic effects mediated through CD4 and CD8 Treg, inhibition of effector T cells and pro-immune cytokines, enhancement of immune-inhibitory cytokines and FOXP3, and absence of DSA. These were even observed in fully allogenic C3H IUT following maternal DC depletion. Additionally, we found that functional tolerance was maintained in DC-depleted pIUT recipients with diminishing and low PB donor and maternal chimerism (< 1%) at the 12th week postnatal boost, whereas in our earlier work pIUT recipients of postnatal boost at 4w were chimeras (DCC^PB^ > 1%). This data supports the findings of Chen et al. that persistent DCC is irrelevant to the maintenance of donor-specific tolerance [43], and suggests that maternal DC suppression prolongs host tolerance acquired from IUT even in the absence of sustained DCC^PB^. We conclude from these data that DCC appears to be independent of the degree of MMc. Maternal DC depletion has no impact on DCC and promotes a tolerogenic response through both Treg and Breg activity by influencing cytokine expression. Both maternal and recipient immune cells respond to donor cells, but only maternal TCR and BCR diversity is affected by maternal DC depletion.

While earlier murine IUT studies concluded that DCC facilitates initial tolerance induction by diminishing donor-specific T-cell and NK cell alloresponsiveness, postulated to occur through Treg augmentation or NK surface receptor downregulation in F1 progeny [43, 44] these models utilised completely allogenic transplantations, whereas our robust semi-allogenic model more accurately reflects the clinical IUT protocol for alpha-thalassaemia major that uses haploidentical maternal bone marrow HSC (clinical trial NCT02986698) and is the first to explore the role of trafficked maternal cells. Our study’s main limitations include the lack of skin grafting to interrogate acquired tolerance in IUT recipients; we instead performed postnatal boost without immunosuppression for this functional assessment. Though we demonstrated donor-specific humoral and cellular hyporesponsiveness to donor cells of both parental origins, postnatal transplantation was only performed on pIUT recipients due to time and cost restrictions. We do not have MLR or TCR/BCR gene expression data from challenged animals. Postnatal transplantations in other IUT recipients and with larger cell doses would have provided useful comparison data regarding the functional effects of the immunomodulatory changes we describe. DCC and immune response may be strain-related [45, 46], hence performing IUT in hybrid offspring of B6 males and BALB/c females would have been informative, though we previously reported that this breeding pattern did not yield differences in maternal microchimerism [8]. We did not explore the role of recipient NK cells in maintaining acquired tolerance, important as these cells undergo adaptation of their Ly49 receptors upon donor antigen exposure and influence DCC [45], though we did not observe differences in recipient NK levels following maternal DC suppression or postnatal challenge.

Inducing the appropriate balance of Th1/Th17/Th2, which influence allograft tolerance or rejection [47], may be critical to improving IUT transplantation tolerance. Maternal TCR/BCR clonotypes displayed substantially reduced diversity post-IUT, restored with DC depletion, while recipient-derived TCR enrichment was unaffected. Our findings indicate that DCC and MMc are separate yet equally important determinants of IUT effectiveness. pIUT produced the highest and most persistent DCC. In contrast to other reported models using maternal donor cells [48, 49], we observed microchimerism post-mIUT despite reduced MMc, similar to our earlier work [8]. Thus, DCC appears primarily dependent on cell origin, not MMc. We demonstrated that maternal cell trafficking is an active process, the quality of which appears to influence the fetal recipient’s immune response to donor cells. Taken together, pIUT resulted in both highest DCC and lowest MMc, and maternal DC depletion further dampened recipient alloresponsiveness through upregulated Treg, the possible presence of Breg, and downregulated proimmune Th cells. aIUT produced both poorest DCC and most robust alloresponsiveness, contrasting with data from other IUT models in which allogenic donor cells have a competitive advantage [50–54]. Although reduced MMc was associated with increased Treg and Breg cytokine expression, upregulated Teff and an overwhelmingly proimmune cytokine response were present, expediting allogeneic cell rejection. Maternal DC may also present donor antigen to recipient immune cells, influencing the quality of immune response. Enhanced Treg production following IUT, particularly of CD62L+ CD25+ FOXP3+ Tregs, protects against graft-v-host disease [55, 56], and together with putative Breg (expressing IL10, IL5, IL6, FOXP3, TGF-β), probably influenced the resulting tolerogenic or immunogenic responses [37, 38]. Human transplantation data indicate that persistent recipient cells inhibit donor haematopoiesis and TCR reconstitution [57–59]. IUT adds an interesting dimension to this, wherein the maternal immune system is the other”recipient” interacting with donor cells, and both maternal and recipient immune systems may impact long-term engraftment and maintenance of tolerance.

Trafficked maternal TCR and BCR clonotypes showing reduced diversity, variable CDR3 lengths and V- and J-segment usage indicate dynamic acquisition of allo-specificity, further underscoring the “sensitization” of maternal cells to transplanted donor cells. Donor cells transplanted directly into the fetus may leak into maternal circulation and participate in maternal immune modelling, selecting and expanding particular clonotypes for trafficking, similar to native fetal microchimerism [60]. The return of DC-depleted maternal clonotypes to higher baseline diversity supports this postulation. Increased recipient TCR diversity confirms that treated fetuses mount antigenic responses even to haploidentical cells, and the diversity remains the same in the DC-depleted recipients, suggesting that the fetuses are less affected by the DT treatment to the pregnant mother. Clinically-poor responders to postnatal hematopoietic stem cell transplantation show lower TCR diversity [61, 62], thus recipient TCR repertoire may be a useful biomarker of transplant rejection [63].

Our murine model permitted specific depletion of antigen-presenting DC in pregnant mice, for which there are no published data. DC depletion at E13 would have endured for the remainder of the pregnancy (parturition ~ E20), resulting in naïve maternal DC trafficked to fetal recipients. Of particular interest is depletion of uterine DC, a unique subtype critical to pregnancy-related tolerance and rejection [12], capable of eliminating fetal cells, which concentrate in maternal circulation towards parturition [60], while not rejecting the fetus [64, 65]. Allogenic fetal cells presented by uterine DC can prime maternal tolerance towards paternal antigens via expansion or elimination of alloreactive or regulatory lymphocytes [66–68]. Donor cells transplanted in mid-gestation likely leak into maternal circulation, prompting reciprocal trafficking of primed maternal cells. Indeed, with DC depletion, maternal TCR clonotypes showed markedly greater diversity, and pIUT and mIUT recipients expanded Treg subsets and upregulated less immunogenic cytokines. Further investigation into uterine DC may intriguingly reveal a unique target for in-vivo or ex-vivo modulation to enhance transplantation tolerance.

Our data raise the exciting possibility of utilising unique maternal and recipient TCR/BCR repertoires in multiple fashion for IUT, and by extension other cellular fetal therapies, by serving as biomarkers of engraftment and immune tolerance, as therapeutic targets to improve transplantation tolerance, e.g. by transducing regulatory sequences into high avidity clonotypes, and as prognosticators of transplantation outcomes. Robust graft tolerance required to maintain life-long engraftment carries several layers [69] that, in combination, may even permit repeat transplantation, including limiting maturation of high-avidity alloreactive T-cells and expanding high-avidity Treg [70–72]. Selective expansion of regulatory TCR clonotypes with distinctly tolerogenic phenotypes may be an individualizable strategy through which to achieve such tolerance [73]. Bregs maintain immune tolerance, but are more difficult to identify due to the absence of unique markers [74, 75]. Though maternal and recipient TCR clonotypes underwent the greatest expansion, we observed an increase in recipient-derived BCR clonotypes along with upregulated Breg-associated cytokines. This suggests that putative regulatory BCR clonotypes are represented and may be valuable components of IUT tolerance. Our data may influence clinical management even in the short-term, supporting the transplantation of paternal donor cells in clinical trials or short-term maternal immune-suppression at the time of IUT, similar to immunotherapy for recurrent miscarriages [76]. Our work is limited by transplantation of a small donor inoculum and limited monitoring for 16 weeks, while pandemic restrictions curtailed our assessment of DC-depletion mIUT and aIUT immune repertoires. Additionally, in the limited number of mIUT and aIUT recipients harvested, donor chimerism was very low which may affect accuracy of donor cell analyses. Transplantation of larger doses to achieve therapeutic DCC and a longer surveillance of maternal/recipient immune profiles in larger animals will be informative for future clinical translation.

## Conclusions

Our results demonstrate that donor cell chimerism depends on donor cell origin, is not influenced by trafficked maternal cells, and maternal and recipient alloresponsiveness can be manipulated via maternal dendritic cell depletion. Transient maternal dendritic cell suppression reduces fetal alloresponsiveness to semi-allogenic donor cells in-utero, instigates selective trafficking of maternal cells, and promotes fetal tolerance. Unique maternal and recipient TCR/BCR repertoires may serve as therapeutic targets to improve transplantation outcomes. This clinically relevant data, similar to our previous study [8], supports paternal IUT for clinical trials and encourages short-term maternal immune suppression at IUT.

## Supplementary Information

**Additional file1**. **Figure S1**. Diphtheria toxin transiently suppresses conventional dendritic cells in various organs, and engrafted donor cells and IUT recipients maintain haemopoietic stem and progenitor markers. **Figure S2.** Intrauterine transplantation elicits active and selective maternal cell trafficking and does not produce donor-specific antibodies. **Table S1**. Antibody conjugates used for flow cytometry. Each antibody was validated with respective positive control as per manufacturer’s instructions. **Table S2**. List of forward and reverse primers used for cytokines and FoxP3 gene expression by qPCR. **Table S3a**. Calculated raw data representing immune profile of DC-depleted pIUT donor cells, maternal immune cells,and recipient cells in BM and PB. Data represents mean ± SD, analysed by two-way ANOVA with Tukey’s multiple comparisons test. Representative bar graphs are displayed in Fig. 2a–f. **Table S3b**. Calculated raw data representing immune profile of DC control pIUT donor cells, maternal immune cells and recipient cells in BM and PB. Data represents mean ± SD, analysed by two-way ANOVA with Tukey’s multiple comparisons test. Representative bar graphs are displayed in Fig. 2a–f. **Table S4a.** Calculated raw data representing immune profile of DC-depleted mIUT donor cells, maternal immune cells and recipient cells in BM and PB. Data represents mean± SD, analysed by two-way ANOVA with Tukey’s multiple comparisons test.  Representative bar graphs are displayed inFig. 3a–f. **Table S4b**. Calculated raw data representing immune profile of DC control mIUT donor cells, maternal immune cells and recipient cells in BM and PB. Data represents mean ± SD, analysed by two-way ANOVA with Tukey’s multiple comparisons test.  Representative bar graphs are displayed inFig. 3a–f. **Table S5a**. Calculated raw data representing immune profile of DC-depleted aIUT donor cells, maternal immune cells and recipient cells in BM and PB. Data represents mean ± SD, analysed by two-way ANOVA with Tukey’s multiple comparisons test.  Representative bar graphs are displayed in Fig.4a–f. **Table S5b**. Calculated raw data representing immune profile of DC control aIUT donor cells, maternal immune cells and recipient cells in BM and PB. Data represents mean ± SD, analysed by two-way ANOVA with Tukey’s multiple comparisons test.  Representative bar graphs are displayed in Fig.4a–f.

## Data Availability

The datasets generated and/or analysed during this study are available from the corresponding author on reasonable request. Correspondence and requests for materials should be addressed to Dr. Citra NZ Mattar.

## Abbreviations

aIUT: Allogenic donor cells (C3H) used in IUT
ANOVA: Analysis of variance
B6: C57BL/6 black mice
BCR: B-cell receptor
BM: Bone marrow
Breg: Regulatory B cells
C3H: C3H/HeNTac mouse strain
cDC: Conventional dendritic cell
CDR3: Complementarity-determining region 3
Central Tm: Central memory T cells (CD44+ CD62L+)
DC: Dendritic cells
DC-depleted: Transient DC depletion in the dam using diphtheria toxin injection before IUT
DC control: Dams without DC depletion prior to IUT; saline injected controls
DCC: Donor cell chimerism/donor cell engraftment
DCC^BM^: Donor cell chimerism/donor cell engraftment in bone marrow
DCC^PB^: Donor cell chimerism/donor cell engraftment in peripheral blood
DSA: Donor-specific antibodies
DT: Diphtheria toxin
DTR: Diphtheria toxin receptor
E13: Embryonic day 13
E14: Embryonic day 14
Effector Tm: Effector memory T cells (CD44+)
F1: Filial 1 generation
FACS: Fluorescence activated cell sorting
FBS: Fetal bovine serum
GAPDH: Glyceraldehyde 3-phosphate dehydrogenase/housekeeping gene
HSC: Hematopoietic stem cells
IGH: Immunoglobulin heavy chain
IGK: Immunoglobulin heavy *Kappa*
IGL: Immunoglobulin heavy *Lambda*
IUT: Intrauterine hematopoietic cell transplantation
LSK: Lin^−^ Sca1^+^ c-Kit^+^
MACS: Magnetic activated cell sorting
MAPK: Mitogen-activated protein kinases
MHC: Major histocompatibility complex
mIUT: Maternal donor cells used in IUT
MLR: Mixed lymphocyte reactivity assay
MMc: Maternal microchimerism—maternal cells trafficked to the fetus
MNC: Mononuclear cells
NK: Natural killer cells
PB: Peripheral blood
qPCR: Quantitative polymerase chain reaction
pIUT: Paternal donor cells used in IUT
SD: Standard deviation
TCR: T-cell receptor
Teff: Effector T cells (CD25+)
TRA: T-cell receptor—Alpha
TRB: T-cell receptor—Beta
TRD: T-cell receptor—Delta
Treg: Regulatory T cells (FOXP3+)
TRG: T-cell receptor—Gamma
VDJ: Variable (V), diversity (D), and joining (J) gene segment
