# Supplementary material for: Maternal dendritic cells influence fetal allograft response following murine in-utero hematopoietic stem cell transplantation
Source: Stem Cell Res Ther. 2023 May 24;14:136. doi: 10.1186/s13287-023-03366-9 (PMC10206581; doi:10.1186/s13287-023-03366-9)
Supplement: Supplementary file 1 — Additional file1. Figure S1. Diphtheria toxin transiently suppresses conventional dendritic cells in various organs, and engrafted donor cells and IUT recipients maintain haemopoietic stem and progenitor markers. Figure S2. Intrauterine transplantation elicits active and selective maternal cell trafficking and does not produce donor-specific antibodies. Table S1. Antibody conjugates used for flow cytometry. Each antibody was validated with respective positive control as per manufacturer’s instructions. Table S2. List of forward and reverse primers used for cytokines and FoxP3 gene expression by qPCR. Table S3a. Calculated raw data representing immune profile of DC-depleted pIUT donor cells, maternal immune cells,and recipient cells in BM and PB. Data represents mean ± SD, analysed by two-way ANOVA with Tukey’s multiple comparisons test. Representative bar graphs are displayed in Fig. 2a–f. Table S3b. Calculated raw data representing immune profile of DC control pIUT donor cells, maternal immune cells and recipient cells in BM and PB. Data represents mean ± SD, analysed by two-way ANOVA with Tukey’s multiple comparisons test. Representative bar graphs are displayed in Fig. 2a–f. Table S4a. Calculated raw data representing immune profile of DC-depleted mIUT donor cells, maternal immune cells and recipient cells in BM and PB. Data represents mean± SD, analysed by two-way ANOVA with Tukey’s multiple comparisons test. Representative bar graphs are displayed inFig. 3a–f. Table S4b. Calculated raw data representing immune profile of DC control mIUT donor cells, maternal immune cells and recipient cells in BM and PB. Data represents mean ± SD, analysed by two-way ANOVA with Tukey’s multiple comparisons test. Representative bar graphs are displayed inFig. 3a–f. Table S5a. Calculated raw data representing immune profile of DC-depleted aIUT donor cells, maternal immune cells and recipient cells in BM and PB. Data represents mean ± SD, analysed by two-way ANOVA with Tukey’s mul [file 13287_2023_3366_MOESM1_ESM.docx]

**Supplemental Data**

**Maternal Dendritic Cells Influence Fetal Allograft Response following Murine In-Utero Hematopoietic Stem Cell Transplantation**

Karthikeyan Kandasamy^1^, Nuryanti Binti Johana^3^, Lay Geok Tan^1,2^, Yvonne Tan^3^, Julie Yeo Su Li^3^, Nur Nazneen Binte Yusof^1^, Li Zhihui^5^, Koh Jiayu^5^, Florent Ginhoux^6,7,8^, Jerry KY Chan^1,3,4^, Mahesh Choolani^1,2^, Citra NZ Mattar^1,2,*^

1. Experimental Fetal Medicine Group, Department of Obstetrics and Gynaecology, Yong Loo Lin School of Medicine, National University of Singapore, 119228 Singapore
2. Department of Obstetrics and Gynaecology, National University Hospital, National University Health System, Singapore
3. Reproductive Medicine, KK Women's and Children's Hospital, Singapore
4. Cancer and Stem Cell Biology Program, Duke-NUS Graduate Medical School, Singapore
5. Genome Research Informatics & Data Science Platform, Genome Institute of Singapore, Agency for Science Technology and Research, Singapore
6. Singapore Immunology Network (SIgN), Agency for Science, Technology and Research (A*STAR), Singapore
7. Translational Immunology Institute, Singhealth/Duke-NUS Academic Medical Centre, the Academia, Singapore
8. Shanghai Institute of Immunology, Shanghai JiaoTong University School of Medicine, Shanghai, China

*Corresponding author: Dr Citra NZ Mattar, Associate Professor, Experimental Fetal Medicine Group, Department of Obstetrics and Gynaecology, Yong Loo Lin School of Medicine, National University of Singapore, 1E Kent Ridge Road Singapore 119228, Telephone: +65-67722672, Fax: +65 6779 4753,

Email: [citramattar@nus.edu.sg](mailto:citramattar@nus.edu.sg)

**Supplemental data – Figure S1**

**
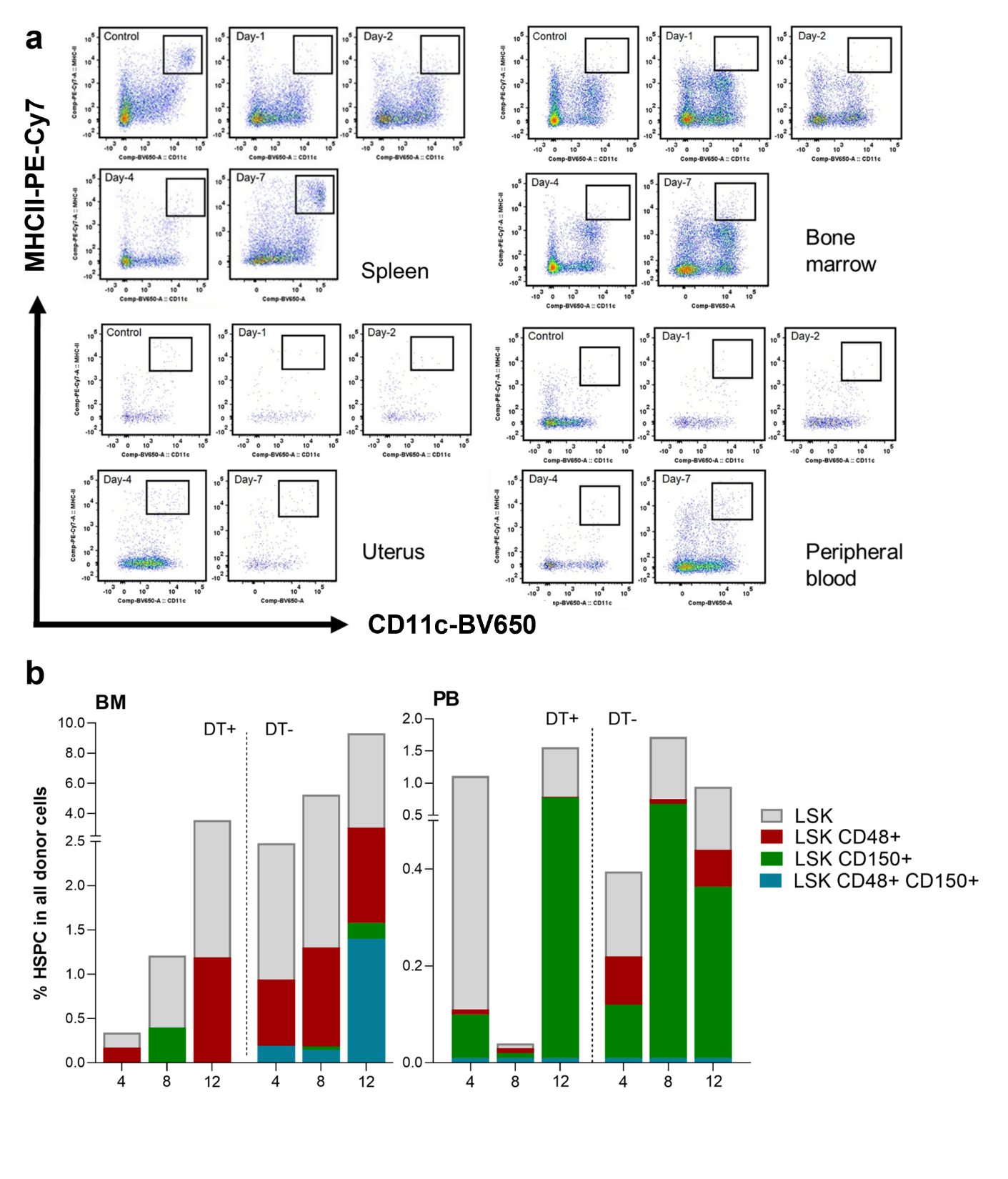
**

**b**

**
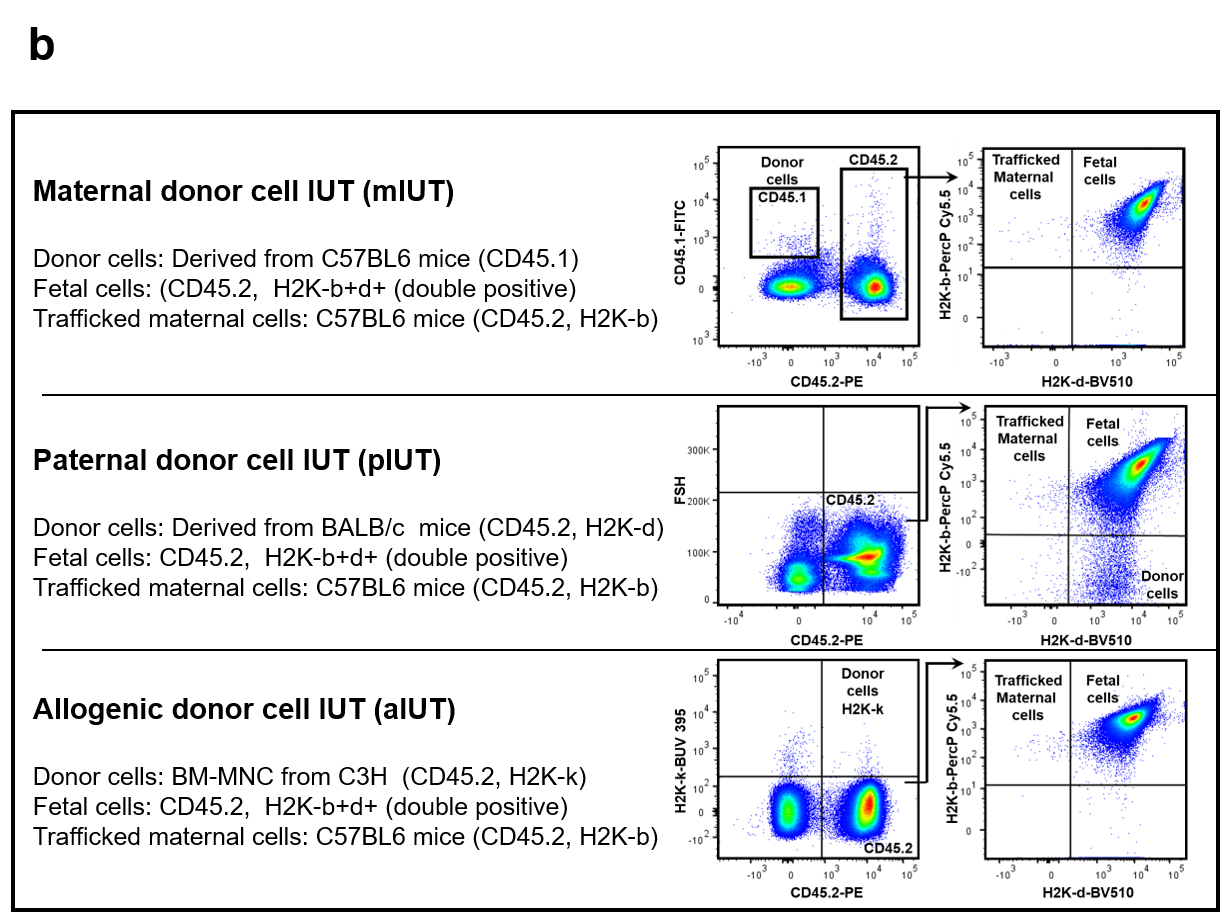
**

**c**

**
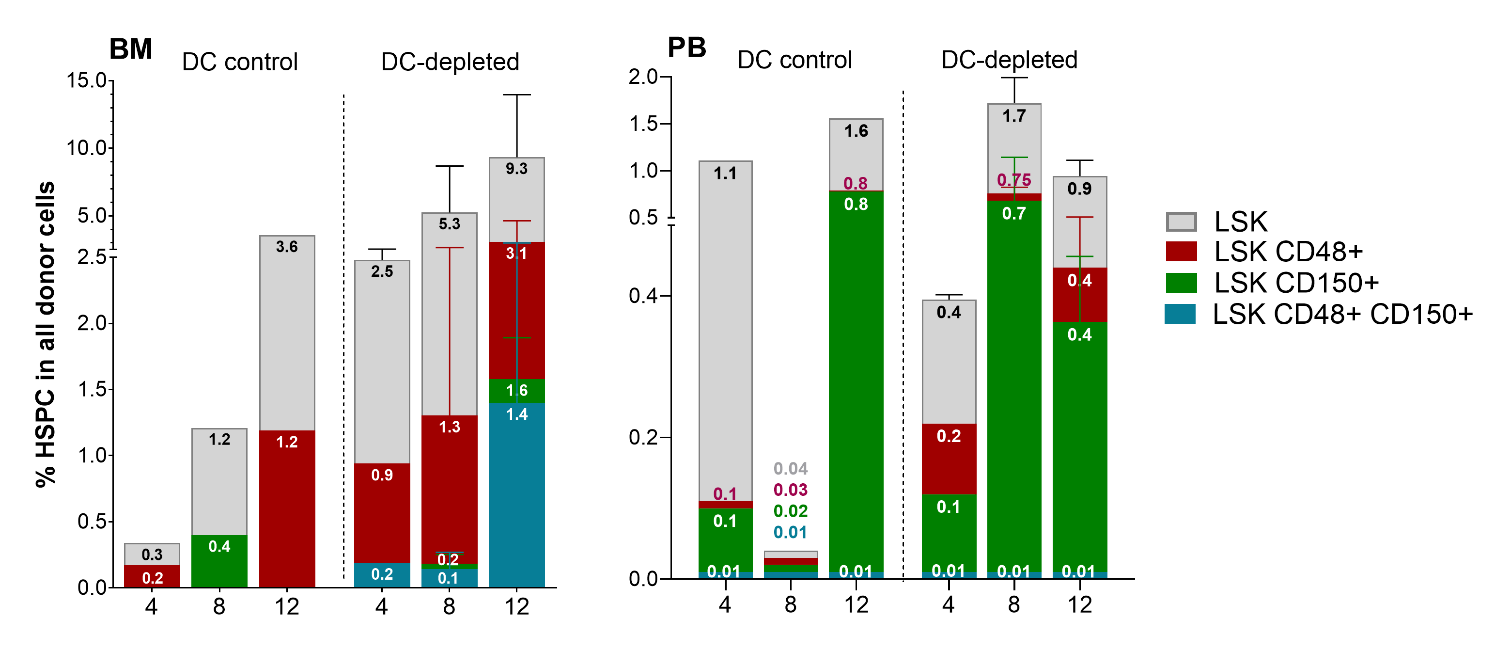
**

**
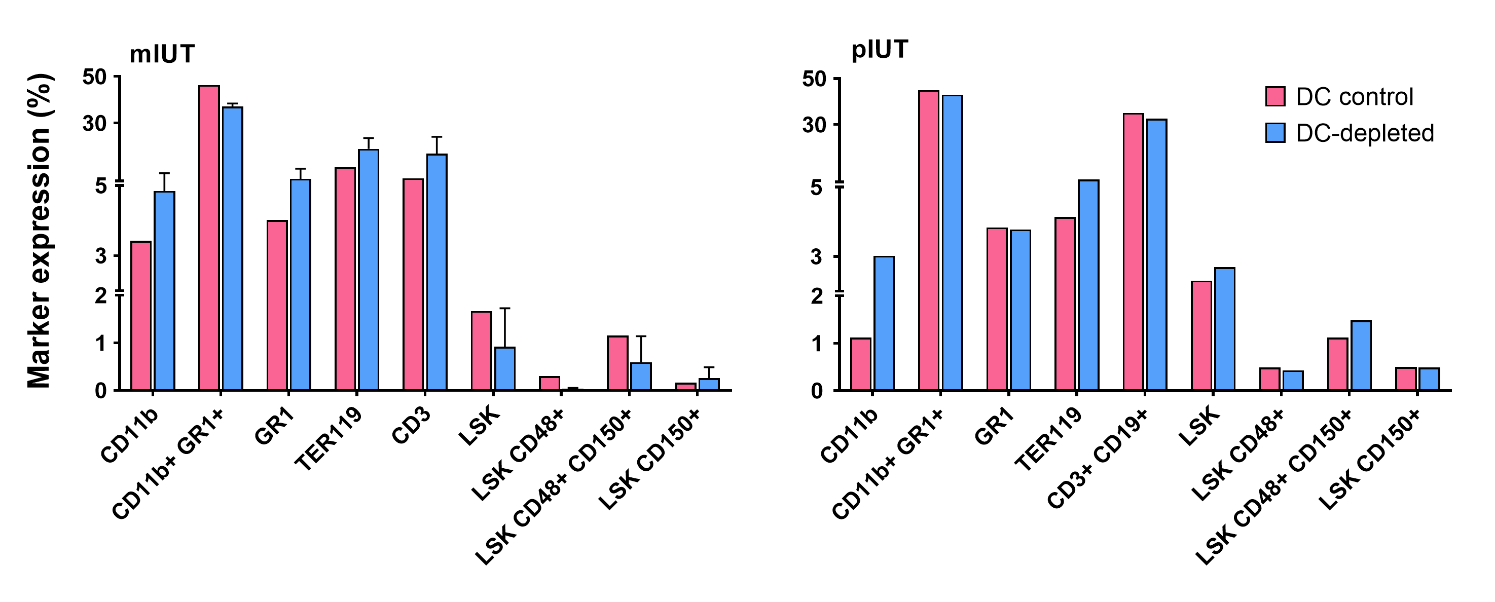
**

**d**

**Supplemental Figure S1. Diphtheria toxin transiently suppresses conventional dendritic cells in various organs, and engrafted donor cells maintain haemopoietic stem and progenitor markers.** (a) Conventional dendritic cells identified as CD11c+ and MHC-II+ (inner box) were observed to be suppressed for up to one week in multiple organs. (b) Flow cytometric identification of donor cells, trafficked maternal cells, and fetal cells in the recipient cells from each IUT experiments. (c) Engrafted donor cells recovered from pIUT recipient bone marrow of DC control (n=9) and DC-depleted (n=11) comprised of LSK (1.1% and 3.9% of total cells), LSK CD48+ (HSC progenitors, 0.5% and 1.1%) and LSK CD150+ (HSC long term repopulation) 1% in both groups respectively. In peripheral blood, DC-depleted (n=8) and DC control (n=8) pIUT recipients shows LSK population (~0.5% in both groups), LSK CD48+ (0.01-0.08%) and LSK CD150+ (0.3%) in both groups respectively. (d) Fetal bone marrow cells from pIUT (week-4) and mIUT (week-0) recipients shows expression of HSC lineage markers (CD11b, GR1, TER119, CD3) and stemness markers (LSK, LSK CD48+, LSK CD48+ CD150+, LSK CD150+) in DC control and DC-depleted groups were similar. Markers CD11b: Monocyte/Macrophages, GR1: Granulocytes, CD11b+GR1+: Myeloid lineage, TER119: Erythroid lineage, CD3: T cells, CD19: B-cells, LSK: Lin-SCA1+ c-Kit+.

**Supplemental data – Figure S2**

**a**

**
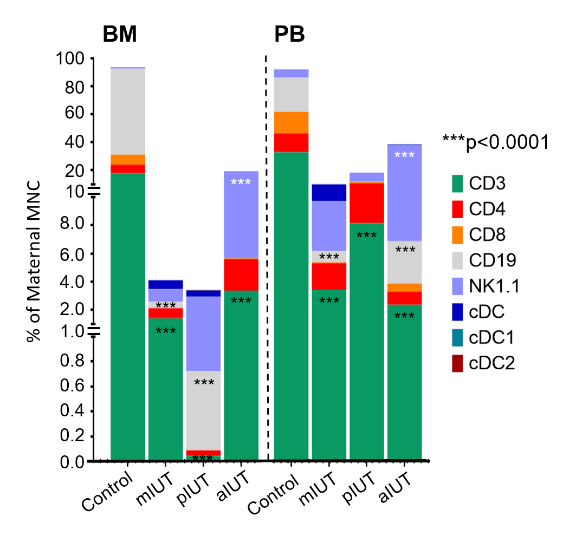
**

**
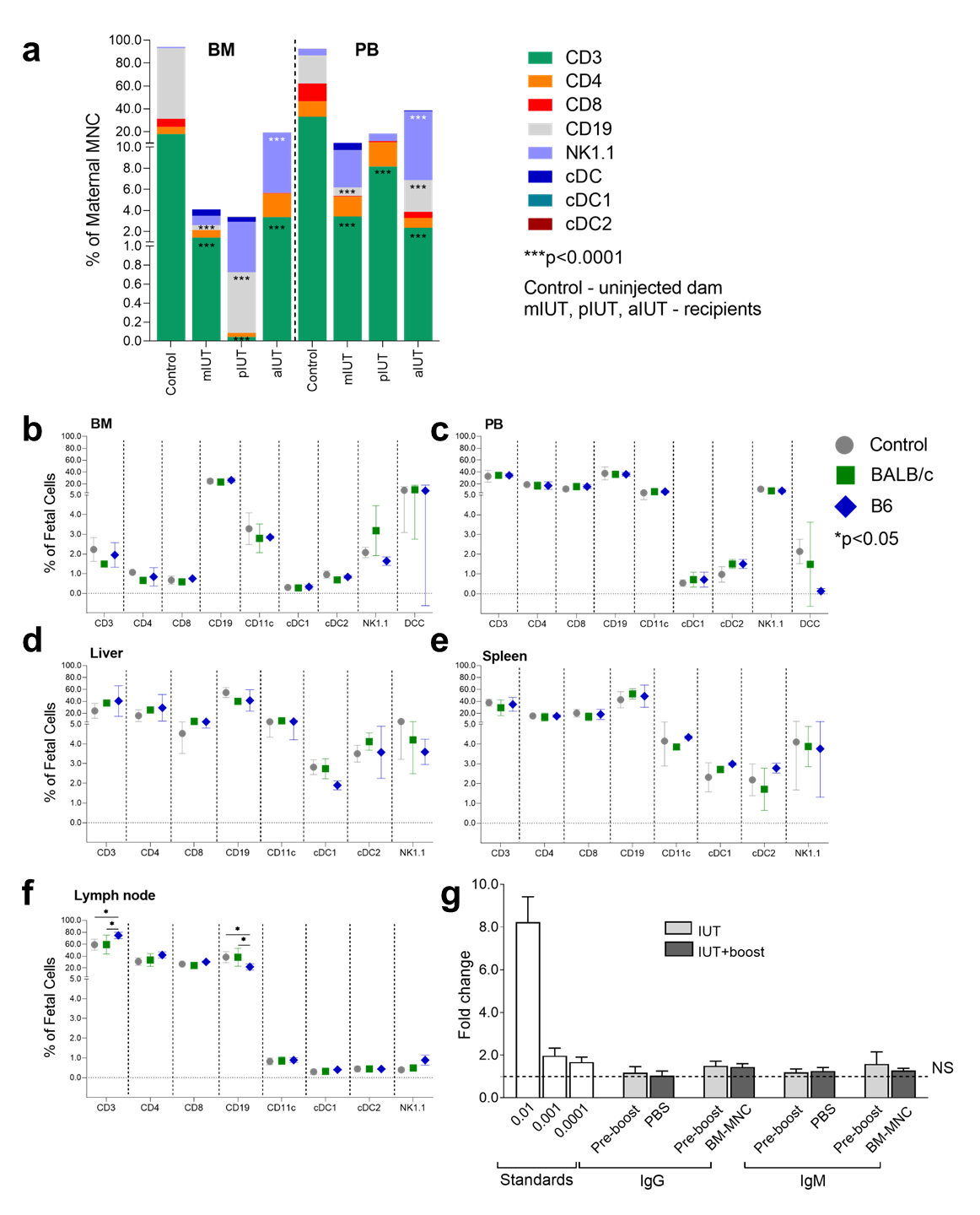
**

**Supplemental Figure S2. Intrauterine transplantation elicits active and selective maternal cell trafficking and does not produce donor-specific antibodies.** Maternal cells recovered from recipient BM and PB had lower levels of all immune cells after any IUT, particularly CD3 and CD19 (black*), compared to the immune profile of non-IUT dams (controls; uninjected pups), except for NK1.1 being higher after aIUT than controls (white*), demonstrating an active process (a). DC-depleted pIUT recipients showed only increased CD3 and reduced CD19 within lymph nodes following B6 boost (b-f, n=7). Donor-specific IgG and IgM were not detected following IUT with/without boost with paternal donor cells (g). Data represent mean ± SD, analysed by two-way ANOVA with Tukey’s multiple comparisons

**Supplemental Table S1: Antibody conjugates used for flow cytometry. Each antibody was validated with respective positive control as per manufacturer’s instructions.**

| **Antibodies** | **Clone** | **Fluorescence** | **Catalogue #** | **Company** |
| --- | --- | --- | --- | --- |
| B220 | RA3-6B2 | APC | 103212 | Biolegend |
| CD117 (c-kit) | 2B8 | BV605 | 563146 | BD Biosciences |
| CD11b | M1/70 | Alexa Fluor 700 | 557960 | BD Biosciences |
| CD11c | N418 | BV650 | 117339 | Biolegend |
| CD127 | SB/199 | BV510 | 563353 | BD Biosciences |
| CD150 | TC15-12F12.2 | BV650 | 115932 | Biolegend |
| CD172a | P84 | Alexa Fluor 700 | 144021 | Biolegend |
| CD19 | 6D5 | FITC | 115506 | Biolegend |
| CD25 | PC61 | BV650 | 564021 | BD Biosciences |
| CD3 | 17A2 | BV421 | 564008 | BD Biosciences |
| CD4 | GK1.5 | Alexa Fluor 700 | 100430 | Biolegend |
| CD44 | IM7 | FITC | 561859 | BD Biosciences |
| CD45.1 | A20 | APC | 110714 | Biolegend |
| CD45.2 | 104 | PE | 109808 | Biolegend |
| CD62L | MEL-14 | BV605 | 563252 | BD Biosciences |
| CD8a | 53-6.7 | BV605 | 563152 | BD Biosciences |
| FC block | 2.4G2 |  | 553142 | BD Biosciences |
| Fixable viability stain 620 | | PE-CF594 | 564996 | BD Biosciences |
| FOXP3 | R16-715 | PerCP Cy5.5 | 563902 | BD Biosciences |
| GR1 | RB6-8C5 | PE-Cy7 | 25593182 | Thermofisher Scientific |
| H-2Kb | AF6-88.5 | PerCP-Cy5.5 | 562831 | BD Biosciences |
| H-2Kd | SF1-1.11 | BV510 | 742432 | BD Biosciences |
| H2K-k | 36-7-5 | BUV395 | 750340 | BD Biosciences |
| IgG | Poly4053 | APC | 405308 | Biolegend |
| IgM | RMM-1 | BV421 | 406517 | Biolegend |
| MHC-II | M5/114.15.2 | PE-Cy7 | 25532182 | Thermofisher Scientific |
| NK1.1 | PK136 | APC-Cy7 | 560618 | BD Biosciences |
| Sca1 | D7 | PerCP | 108121 | Biolegend |
| TER119 | TER-119 | APC-Cy7 | 560509 | BD Biosciences |
| XCR1 | ZET | APC-Cy7 | 148223 | Biolegend |

**Supplemental data: Table S2: List of forward and reverse primers used for cytokines and FoxP3 gene expression by qPCR**

| **Acronym/ Gene ID** | **Strand** | **Primer Pairs** | **Amplicon (bp)** |
| --- | --- | --- | --- |
| **IL-1β** | Forward | 5'-AGTTGACGGACCCCAAAAGAT-3' | 90 |
|  | Reverse | 5'-GTGCTGCTGCGAGATTTGAA-3' |  |
| **IL-1rn** | Forward | 5'-CCACCACCAGCTTTGAGTCA-3' | 70 |
|  | Reverse | 5'-GGACGGTCAGCCTCTAGTGTTG-3' |  |
| **IL-2** | Forward | 5'-CCTGAGCAGGATGGAGAATTACA-3' | 132 |
|  | Reverse | 5'-TGCCGCAGAGGTCCAAGT-3' |  |
| **IL-4** | Forward | 5'-ACCCCCAGCTAGTTGTCATCCT-3' | 70 |
|  | Reverse | 5'-TTGTCGCATCCGTGGATATG-3' |  |
| **IL-5** | Forward | 5'-CCATGAGCACAGTGGTGAAAGA-3' | 80 |
|  | Reverse | 5'-CCTCATCGTCTCATTGCTTGTC-3' |  |
| **IL-6** | Forward | 5'-CCAACAGACCTGTCTATACCACTTCA-3' | 70 |
|  | Reverse | 5'-TCCACGATTTCCCAGAGAACA-3' |  |
| **IL-7** | Forward | 5'-GTGCTGCTCGCAAGTTGAAG-3' | 93 |
|  | Reverse | 5'-GTGTTTGTGTGCCTTGTGATACTG-3' |  |
| **IL-10** | Forward | 5'-GCCAGTACAGCCGGGAAGA-3' | 83 |
|  | Reverse | 5'-GAAGGCAGTCCGCAGCTCTA-3' |  |
| **IL-12a** | Forward | 5'-ATCAACGCAGCACTTCAGAATC-3' | 113 |
|  | Reverse | 5'-CGCAGAGTCTCGCCATTATG-3' |  |
| **IL-12b** | Forward | 5'-GACTCTCGGGCAGTGACATGT-3' | 70 |
|  | Reverse | 5'-AGTCCCTTTGGTCCAGTGTGA-3' |  |
| **IL-13** | Forward | 5'-TTCCCTGACCAACATCTCCAA-3' | 70 |
|  | Reverse | 5'-GTTACAGAGGCCATGCAATATCC-3' |  |
| **IL-17a** | Forward | 5'-ACCGCAATGAAGACCCTGAT-3' | 84 |
|  | Reverse | 5'-TTCCCTCCGCATTGACACA-3' |  |
| **IL-17f** | Forward | 5'-CTTGCAGAAGGCTGGGAACT-3' | 79 |
|  | Reverse | 5'-CTGGTTTTGGTTGAAGATTCGAA-3' |  |
| **IL-21** | Forward | 5'-TGAAAATGACTTGGATCCTGAACTT-3' | 111 |
|  | Reverse | 5'-GGGTTTGATGGCTTGAGTTTG-3' |  |
| **IL-22** | Forward | 5'-CATCGTCAACCGCACCTTTA-3' | 70 |
|  | Reverse | 5'-GAGCCGGACATCTGTGTTGTT-3' |  |
| **CSF-2** | Forward | 5'-GTCACGTTGAATGAAGAGGTAGAAGT-3' | 70 |
|  | Reverse | 5'-TCTGCACACATGTTAGCTTCTTGA-3' |  |
| **FOXP3** | Forward | 5'-CACAACCTGAGCCTGCACAA-3' | 78 |
|  | Reverse | 5'-CTCAAATTCATCTACGGTCCACACT-3' |  |
| **IFN-γ** | Forward | 5'-CTGGAGGAACTGGCAAAAGG-3' | 103 |
|  | Reverse | 5'-GATGGCCTGATTGTCTTTCAAGA-3' |  |
| **TGF-β2** | Forward | 5'-CTGCCTTCGCCCTCTTTACA-3' | 94 |
|  | Reverse | 5'-CCCAGCACAGAAGTTAGCATTG-3' |  |
| **TNF-α** | Forward | 5'-GCCTCTTCTCATTCCTGCTTGT-3' | 71 |
|  | Reverse | 5'-ACCGATCACCCCGAAGTTC-3' |  |
| **GAPDH** | Forward | 5'-GAGTGTTTCCTCGTCCCGTAGA-3' | 75 |
|  | Reverse | 5'-TGACCAGGCGCCCAATAC-3' |  |

**Supplemental data table S3a:** Calculated raw data representing immune profile of DC-depleted pIUT donor cells, maternal immune cells, (MMc) and recipient cells in BM and PB. Data represents mean ± SD, analysed by two-way ANOVA with Tukey’s multiple comparisons test. Representative bar graphs are displayed in figure 2a-f.

|  |  | **Total DCC** | | **CD3** | | **CD4** | | **CD8** | | **CD19** | | **cDC** | | **cDC1** | | **cDC2** | | **NK1.1** | |
| --- | --- | --- | --- | --- | --- | --- | --- | --- | --- | --- | --- | --- | --- | --- | --- | --- | --- | --- | --- |
|  | Weeks | Mean | SD | Mean | SD | Mean | SD | Mean | SD | Mean | SD | Mean | SD | Mean | SD | Mean | SD | Mean | SD |
| Donor immune cells - BM | 0 | 11.86 | 9.38 | 0.85 | 0.49 | 0.38 | 0.26 | 0.02 | 0.26 | 4.49 | 0.35 | 0.02 | 0.01 | 0.01 | 0.02 | 0.00 | 0.00 | 1.90 | 1.44 |
|  | 4 | 2.23 | 1.28 | 0.10 | 0.04 | 0.08 | 0.04 | 0.01 | 0.04 | 9.48 | 6.44 | 0.02 | 0.02 | 0.01 | 0.02 | 0.02 | 0.02 | 0.81 | 0.41 |
|  | 8 | 5.63 | 8.09 | 0.06 | 0.01 | 0.04 | 0.01 | 0.01 | 0.01 | 4.14 | 2.07 | 0.01 | 0.01 | 0.00 | 0.00 | 0.01 | 0.01 | 1.46 | 1.61 |
|  | 12 | 13.84 | 1.17 | 0.03 | 0.02 | 0.02 | 0.01 | 0.00 | 0.01 | 1.31 | 0.19 | 0.02 | 0.01 | 0.01 | 0.01 | 0.01 | 0.01 | 0.91 | 0.25 |
|  |  |  |  |  |  |  |  |  |  |  |  |  |  |  |  |  |  |  |  |
| Donor immune cells - PB | 0 | 8.22 | 5.94 | 6.00 | 5.80 | 1.77 | 0.89 | 0.00 | 0.00 | 22.07 | 15.57 | 0.24 | 0.34 | 0.24 | 0.34 | 0.24 | 0.34 | 3.70 | 3.61 |
|  | 4 | 2.41 | 1.82 | 5.04 | 3.11 | 2.39 | 2.20 | 1.57 | 0.64 | 15.45 | 7.13 | 0.11 | 0.20 | 0.11 | 0.20 | 0.11 | 0.20 | 2.76 | 2.06 |
|  | 8 | 0.53 | 0.91 | 1.99 | 1.60 | 0.98 | 1.08 | 0.57 | 0.06 | 26.39 | 17.26 | 0.18 | 0.31 | 0.09 | 0.16 | 0.09 | 0.16 | 3.89 | 4.72 |
|  | 12 | 0.82 | 0.52 | 2.03 | 1.56 | 1.48 | 1.02 | 0.40 | 0.48 | 12.86 | 0.56 | 0.28 | 0.20 | 0.28 | 0.20 | 0.20 | 0.26 | 2.59 | 0.15 |
|  |  |  |  |  |  |  |  |  |  |  |  |  |  |  |  |  |  |  |  |
|  |  | **Total MMc** | | **CD3** | | **CD4** | | **CD8** | | **CD19** | | **cDC** | | **cDC1** | | **cDC2** | | **NK1.1** | |
|  | Weeks | Mean | SD | Mean | SD | Mean | SD | Mean | SD | Mean | SD | Mean | SD | Mean | SD | Mean | SD | Mean | SD |
| MMc - BM | 0 | 0.01 | 0.01 | 0.00 | 0.00 | 0.00 | 0.00 | 0.00 | 0.00 | 0.00 | 0.00 | 0.00 | 0.00 | 0.00 | 0.00 | 0.00 | 0.00 | 2.50 | 3.54 |
|  | 4 | 0.89 | 0.75 | 0.07 | 0.07 | 0.06 | 0.07 | 0.00 | 0.00 | 0.17 | 0.10 | 0.00 | 0.00 | 0.00 | 0.00 | 0.00 | 0.00 | 1.03 | 0.68 |
|  | 8 | 0.50 | 0.57 | 0.00 | 0.00 | 0.00 | 0.00 | 0.00 | 0.00 | 0.53 | 0.82 | 0.01 | 0.01 | 0.00 | 0.00 | 0.00 | 0.00 | 3.34 | 3.09 |
|  | 12 | 0.52 | 0.42 | 0.09 | 0.05 | 0.01 | 0.02 | 0.08 | 0.07 | 0.01 | 0.02 | 0.00 | 0.00 | 0.00 | 0.00 | 0.00 | 0.00 | 1.88 | 0.54 |
|  |  |  |  |  |  |  |  |  |  |  |  |  |  |  |  |  |  |  |  |
| MMc - PB | 0 | 0.04 | 0.05 | 0.00 | 0.00 | 0.00 | 0.00 | 0.00 | 0.00 | 0.00 | 0.00 | 0.00 | 0.00 | 0.00 | 0.00 | 0.00 | 0.00 | 0.00 | 0.00 |
|  | 4 | 0.96 | 1.11 | 6.12 | 10.61 | 2.04 | 3.53 | 0.68 | 1.18 | 0.46 | 0.79 | 0.00 | 0.00 | 0.00 | 0.00 | 0.00 | 0.00 | 5.53 | 4.07 |
|  | 8 | 0.22 | 0.13 | 0.30 | 0.53 | 0.00 | 0.00 | 0.00 | 0.00 | 1.88 | 1.82 | 0.00 | 0.00 | 0.00 | 0.00 | 0.00 | 0.00 | 5.66 | 3.76 |
|  | 12 | 0.18 | 0.08 | 0.00 | 0.00 | 0.00 | 0.00 | 0.00 | 0.00 | 4.22 | 2.17 | 0.00 | 0.00 | 0.00 | 0.00 | 0.00 | 0.00 | 5.82 | 4.95 |

|  |  |  | | **CD3** | | **CD4** | | **CD8** | | **CD19** | | **cDC** | | **cDC1** | | **cDC2** | | **NK1.1** | |
| --- | --- | --- | --- | --- | --- | --- | --- | --- | --- | --- | --- | --- | --- | --- | --- | --- | --- | --- | --- |
|  | Weeks |  |  | Mean | SD | Mean | SD | Mean | SD | Mean | SD | Mean | SD | Mean | SD | Mean | SD | Mean | SD |
| Recipient immune cells - BM | 0 |  |  | 0.82 | 0.49 | 0.36 | 0.30 | 0.07 | 0.01 | 8.59 | 5.93 | 0.14 | 0.04 | 0.09 | 0.10 | 0.03 | 0.01 | 1.37 | 0.91 |
|  | 4 |  |  | 0.36 | 0.24 | 0.16 | 0.05 | 0.10 | 0.12 | 35.28 | 28.75 | 0.10 | 0.03 | 0.06 | 0.04 | 0.08 | 0.03 | 0.74 | 0.24 |
|  | 8 |  |  | 1.33 | 0.19 | 0.49 | 0.14 | 0.64 | 0.15 | 37.39 | 3.06 | 0.20 | 0.02 | 0.05 | 0.06 | 0.16 | 0.02 | 1.38 | 1.04 |
|  | 12 |  |  | 1.35 | 0.03 | 0.57 | 0.06 | 0.53 | 0.13 | 23.03 | 4.11 | 0.28 | 0.07 | 0.27 | 0.08 | 0.24 | 0.05 | 1.03 | 0.22 |
|  |  |  |  |  |  |  |  |  |  |  |  |  |  |  |  |  |  |  |  |
| Recipient immune cells - PB | 0 |  |  | 1.80 | 0.60 | 8.50 | 3.07 | 1.07 | 0.91 | 20.51 | 0.90 | 0.68 | 0.18 | 0.45 | 0.51 | 0.45 | 0.36 | 1.98 | 0.54 |
|  | 4 |  |  | 10.96 | 9.05 | 23.41 | 18.50 | 11.98 | 9.70 | 11.60 | 5.54 | 0.18 | 0.16 | 0.08 | 0.13 | 0.17 | 0.15 | 2.70 | 1.23 |
|  | 8 |  |  | 23.01 | 8.46 | 29.80 | 9.56 | 18.80 | 1.65 | 21.61 | 5.28 | 0.30 | 0.22 | 0.03 | 0.01 | 0.28 | 0.20 | 7.37 | 2.70 |
|  | 12 |  |  | 19.94 | 5.09 | 19.93 | 0.83 | 14.09 | 0.46 | 34.29 | 6.40 | 0.30 | 0.16 | 0.30 | 0.16 | 0.28 | 0.15 | 9.73 | 0.23 |

**Supplemental data table S3b:** Calculated raw data representing immune profile of DC control pIUT donor cells, maternal immune cells (MMc) and recipient cells in BM and PB. Data represents mean ± SD, analysed by two-way ANOVA with Tukey’s multiple comparisons test. Representative bar graphs are displayed in figure 2a-f.

|  |  | **Total DCC** | | **CD3** | | **CD4** | | **CD8** | | **CD19** | | **cDC** | | **cDC1** | | **cDC2** | | **NK1.1** | |
| --- | --- | --- | --- | --- | --- | --- | --- | --- | --- | --- | --- | --- | --- | --- | --- | --- | --- | --- | --- |
|  | Weeks | Mean | SD | Mean | SD | Mean | SD | Mean | SD | Mean | SD | Mean | SD | Mean | SD | Mean | SD | Mean | SD |
| Donor immune cells - BM | 0 | 22.54 | 2.23 | 1.93 | 1.34 | 0.73 | 0.54 | 0.03 | 0.01 | 2.46 | 0.99 | 0.01 | 0.01 | 0.01 | 0.01 | 0.01 | 0.01 | 4.19 | 4.85 |
|  | 4 | 11.99 | 10.16 | 0.10 | 0.00 | 0.05 | 0.00 | 0.02 | 0.00 | 4.14 | 1.41 | 0.01 | 0.00 | 0.00 | 0.00 | 0.01 | 0.00 | 0.70 | 0.00 |
|  | 8 | 1.97 | 0.23 | 0.03 | 0.00 | 0.03 | 0.00 | 0.00 | 0.00 | 1.48 | 0.68 | 0.01 | 0.00 | 0.00 | 0.00 | 0.01 | 0.00 | 1.27 | 0.00 |
|  | 12 | 14.56 | 12.35 | 0.18 | 0.00 | 0.17 | 0.00 | 0.00 | 0.00 | 0.11 | 0.00 | 0.01 | 0.00 | 0.01 | 0.00 | 0.01 | 0.00 | 0.32 | 0.00 |
|  |  |  |  |  |  |  |  |  |  |  |  |  |  |  |  |  |  |  |  |
| Donor immune cells - PB | 0 | 13.68 | 4.50 | 4.37 | 2.11 | 2.69 | 1.85 | 0.74 | 0.36 | 28.12 | 17.66 | 0.30 | 0.42 | 0.30 | 0.42 | 0.56 | 0.79 | 0.78 | 0.16 |
|  | 4 | 4.46 | 0.15 | 9.52 | 0.00 | 4.05 | 0.00 | 0.01 | 0.00 | 72.06 | 2.91 | 0.00 | 0.00 | 0.00 | 0.00 | 0.00 | 0.00 | 4.55 | 0.77 |
|  | 8 | 4.19 | 0.02 | 0.44 | 0.00 | 0.35 | 0.00 | 0.01 | 0.01 | 1.23 | 0.00 | 0.00 | 0.00 | 0.00 | 0.00 | 0.00 | 0.00 | 1.70 | 0.00 |
|  | 12 | 0.97 | 0.04 | 0.88 | 0.00 | 0.71 | 0.00 | 0.01 | 0.01 | 9.36 | 0.00 | 0.19 | 0.01 | 0.20 | 0.00 | 0.15 | 0.07 | 4.56 | 0.79 |
|  |  |  |  |  |  |  |  |  |  |  |  |  |  |  |  |  |  |  |  |
|  |  | **Total MMc** | | **CD3** | | **CD4** | | **CD8** | | **CD19** | | **cDC** | | **cDC1** | | **cDC2** | | **NK1.1** | |
|  | Weeks | Mean | SD | Mean | SD | Mean | SD | Mean | SD | Mean | SD | Mean | SD | Mean | SD | Mean | SD | Mean | SD |
| MMc - BM | 0 | 0.06 | 0.08 | 10.00 | 14.14 | 0.00 | 0.00 | 0.00 | 0.00 | 0.00 | 0.00 | 0.00 | 0.00 | 0.00 | 0.00 | 0.00 | 0.00 | 0.00 | 0.00 |
|  | 4 | 0.59 | 0.00 | 0.05 | 0.00 | 0.05 | 0.00 | 0.00 | 0.00 | 0.10 | 0.00 | 0.00 | 0.00 | 0.00 | 0.00 | 0.00 | 0.00 | 0.19 | 0.00 |
|  | 8 | 0.31 | 0.00 | 0.17 | 0.00 | 0.09 | 0.00 | 0.09 | 0.00 | 1.47 | 0.00 | 0.00 | 0.00 | 0.00 | 0.00 | 0.00 | 0.00 | 1.13 | 0.00 |
|  | 12 | 0.33 | 0.00 | 0.00 | 0.00 | 0.00 | 0.00 | 0.00 | 0.00 | 0.00 | 0.00 | 0.00 | 0.00 | 0.00 | 0.00 | 0.00 | 0.00 | 0.41 | 0.00 |
|  |  |  |  |  |  |  |  |  |  |  |  |  |  |  |  |  |  |  |  |
| MMc - PB | 0 | 0.04 | 0.05 | 0.00 | 0.00 | 0.00 | 0.00 | 0.00 | 0.00 | 0.00 | 0.00 | 0.00 | 0.00 | 0.00 | 0.00 | 0.00 | 0.00 | 0.00 | 0.00 |
|  | 4 | 3.12 | 0.00 | 0.34 | 0.59 | 0.34 | 0.59 | 0.00 | 0.00 | 0.00 | 0.00 | 0.00 | 0.00 | 0.00 | 0.00 | 0.00 | 0.00 | 3.27 | 5.66 |
|  | 8 | 0.63 | 0.00 | 0.00 | 0.00 | 0.00 | 0.00 | 0.00 | 0.00 | 0.27 | 0.00 | 0.00 | 0.00 | 0.00 | 0.00 | 0.00 | 0.00 | 0.72 | 1.25 |
|  | 12 | 0.11 | 0.00 | 0.51 | 0.88 | 0.51 | 0.88 | 0.00 | 0.00 | 0.00 | 0.00 | 0.00 | 0.00 | 0.00 | 0.00 | 0.00 | 0.00 | 0.56 | 0.96 |

|  |  |  | | **CD3** | | **CD4** | | **CD8** | | **CD19** | | **cDC** | | **cDC1** | | **cDC2** | | **NK1.1** | | |
| --- | --- | --- | --- | --- | --- | --- | --- | --- | --- | --- | --- | --- | --- | --- | --- | --- | --- | --- | --- | --- |
|  | Weeks |  |  | Mean | SD | Mean | SD | Mean | SD | Mean | SD | Mean | SD | Mean | SD | Mean | SD | Mean | SD |  |
| Recipient immune cells - BM | 0 |  |  | 1.96 | 1.27 | 0.91 | 0.74 | 0.08 | 0.01 | 6.20 | 1.01 | 0.16 | 0.08 | 0.15 | 0.10 | 0.07 | 0.02 | 2.89 | 3.40 |  |
|  | 4 |  |  | 0.60 | 0.00 | 0.21 | 0.00 | 0.22 | 0.00 | 39.18 | 0.00 | 0.15 | 0.00 | 0.02 | 0.00 | 0.11 | 0.00 | 0.84 | 0.00 |  |
|  | 8 |  |  | 2.05 | 0.00 | 0.97 | 0.00 | 0.93 | 0.00 | 37.01 | 0.00 | 0.16 | 0.00 | 0.09 | 0.00 | 0.11 | 0.01 | 1.32 | 0.00 |  |
|  | 12 |  |  | 0.72 | 0.00 | 0.31 | 0.00 | 0.27 | 0.00 | 3.46 | 0.00 | 0.09 | 0.00 | 0.08 | 0.00 | 0.07 | 0.00 | 0.78 | 0.00 |  |
|  |  |  |  |  |  |  |  |  |  |  |  |  |  |  |  |  |  |  |  |  |
| Recipient immune cells - PB | 0 |  |  | 7.24 | 2.06 | 3.95 | 1.36 | 0.99 | 0.57 | 35.99 | 11.68 | 1.05 | 0.59 | 0.96 | 0.66 | 0.38 | 0.20 | 0.59 | 0.20 |  |
|  | 4 |  |  | 12.30 | 0.00 | 5.08 | 0.00 | 0.35 | 0.00 | 71.02 | 0.00 | 0.00 | 0.00 | 0.00 | 0.00 | 0.00 | 0.00 | 3.96 | 0.00 |  |
|  | 8 |  |  | 35.23 | 0.00 | 21.19 | 0.00 | 13.43 | 0.00 | 25.20 | 0.00 | 0.14 | 0.00 | 0.08 | 0.00 | 0.12 | 0.00 | 8.63 | 0.00 |  |
|  | 12 |  |  | 28.52 | 0.00 | 13.97 | 0.00 | 13.90 | 0.00 | 41.82 | 0.00 | 0.26 | 0.00 | 0.26 | 0.00 | 0.23 | 0.00 | 8.49 | 0.00 |  |

**Supplemental data table S4a:** Calculated raw data representing immune profile of DC-depleted mIUT donor cells, maternal immune cells (MMc) and recipient cells in BM and PB. Data represents mean± SD, analysed by two-way ANOVA with Tukey’s multiple comparisons test. Representative bar graphs are displayed in figure 3a-f.

|  |  | **Total DCC** | | **CD3** | | **CD4** | | **CD8** | | **CD19** | | **cDC** | | **cDC1** | | **cDC2** | | **NK1.1** | |
| --- | --- | --- | --- | --- | --- | --- | --- | --- | --- | --- | --- | --- | --- | --- | --- | --- | --- | --- | --- |
|  | Weeks | Mean | SD | Mean | SD | Mean | SD | Mean | SD | Mean | SD | Mean | SD | Mean | SD | Mean | SD | Mean | SD |
| Donor immune cells - BM | 0 | 0.66 | 0.93 | 36.54 | 9.82 | 2.94 | 2.00 | 24.04 | 3.25 | 0.34 | 0.47 | 0.79 | 1.12 | 0.73 | 1.04 | 0.63 | 0.89 | 7.39 | 8.00 |
|  | 4 | 0.01 | 0.00 | 35.29 | 0.00 | 0.00 | 0.00 | 14.71 | 0.00 | 38.24 | 0.00 | 0.00 | 0.00 | 0.00 | 0.00 | 0.00 | 0.00 | 5.88 | 0.00 |
|  | 8 | 0.01 | 0.00 | 62.96 | 0.00 | 0.00 | 0.00 | 14.81 | 0.00 | 1.85 | 0.00 | 0.00 | 0.00 | 0.00 | 0.00 | 0.00 | 0.00 | 5.56 | 0.00 |
|  | 12 | 0.01 | 0.00 | 6.94 | 0.00 | 0.00 | 0.00 | 5.56 | 0.00 | 12.50 | 0.00 | 0.00 | 0.00 | 0.00 | 0.00 | 0.00 | 0.00 | 4.17 | 0.00 |
|  |  |  |  |  |  |  |  |  |  |  |  |  |  |  |  |  |  |  |  |
| Donor immune cells -PB | 4 | 0.01 | 0.01 | 35.87 | 0.00 | 1.09 | 0.00 | 26.09 | 0.00 | 4.35 | 0.00 | 0.00 | 0.00 | 0.00 | 0.00 | 0.00 | 0.00 | 5.43 | 0.00 |
|  | 8 | 0.04 | 0.04 | 11.30 | 0.00 | 2.61 | 0.00 | 1.74 | 0.00 | 6.96 | 0.00 | 0.85 | 0.00 | 0.85 | 0.00 | 0.85 | 0.00 | 6.09 | 0.00 |
|  | 12 | 0.01 | 0.00 | 16.10 | 0.00 | 1.69 | 0.00 | 8.47 | 0.00 | 2.54 | 0.00 | 2.36 | 0.00 | 1.57 | 0.00 | 2.36 | 0.00 | 3.39 | 0.00 |
|  |  |  |  |  |  |  |  |  |  |  |  |  |  |  |  |  |  |  |  |
|  |  | **Total MMc** | | **CD3** | | **CD4** | | **CD8** | | **CD19** | | **cDC** | | **cDC1** | | **cDC2** | | **NK1.1** | |
|  | Weeks | Mean | SD | Mean | SD | Mean | SD | Mean | SD | Mean | SD | Mean | SD | Mean | SD | Mean | SD | Mean | SD |
| MMc - BM | 0 | 0.11 | 0.05 | 2.09 | 2.96 | 1.05 | 1.48 | 0.00 | 0.00 | 0.55 | 0.28 | 0.00 | 0.00 | 0.00 | 0.00 | 0.00 | 0.00 | 0.58 | 0.18 |
|  | 4 | 2.96 | 0.00 | 0.00 | 0.00 | 0.00 | 0.00 | 0.00 | 0.00 | 1.67 | 0.00 | 0.00 | 0.00 | 0.00 | 0.00 | 0.00 | 0.00 | 5.12 | 0.00 |
|  | 8 | 2.16 | 0.00 | 0.06 | 0.00 | 0.01 | 0.00 | 0.02 | 0.00 | 1.98 | 0.00 | 0.03 | 0.00 | 0.00 | 0.00 | 0.00 | 0.00 | 6.18 | 0.00 |
|  | 12 | 0.52 | 0.00 | 0.20 | 0.00 | 0.16 | 0.00 | 0.00 | 0.00 | 0.10 | 0.00 | 0.00 | 0.00 | 0.03 | 0.00 | 0.00 | 0.00 | 6.12 | 0.00 |
|  |  |  |  |  |  |  |  |  |  |  |  |  |  |  |  |  |  |  |  |
| MMc - PB | 4 | 0.64 | 0.00 | 6.86 | 0.00 | 3.79 | 0.00 | 0.12 | 0.00 | 0.25 | 0.00 | 0.01 | 0.00 | 0.01 | 0.00 | 0.00 | 0.00 | 4.36 | 0.00 |
|  | 8 | 0.97 | 0.00 | 9.64 | 0.00 | 3.57 | 0.00 | 3.06 | 0.00 | 2.23 | 0.00 | 0.00 | 0.00 | 0.00 | 0.00 | 0.00 | 0.00 | 44.86 | 0.00 |
|  | 12 | 0.01 | 0.00 | 3.94 | 0.00 | 1.18 | 0.00 | 0.39 | 0.00 | 0.00 | 0.00 | 0.00 | 0.00 | 0.00 | 0.00 | 0.00 | 0.00 | 6.69 | 0.00 |

|  |  |  | | **CD3** | | **CD4** | | **CD8** | | **CD19** | | **cDC** | | **cDC1** | | **cDC2** | | **NK1.1** | | |
| --- | --- | --- | --- | --- | --- | --- | --- | --- | --- | --- | --- | --- | --- | --- | --- | --- | --- | --- | --- | --- |
|  | Weeks |  |  | Mean | SD | Mean | SD | Mean | SD | Mean | SD | Mean | SD | Mean | SD | Mean | SD | Mean | SD |  |
| Recipient immune cells - BM | 0 |  |  | 1.13 | 1.06 | 1.09 | 0.00 | 0.26 | 0.16 | 27.66 | 19.52 | 0.31 | 0.06 | 0.29 | 0.03 | 0.18 | 0.03 | 1.71 | 0.45 |  |
|  | 4 |  |  | 0.62 | 0.00 | 0.20 | 0.00 | 0.24 | 0.00 | 25.24 | 35.69 | 0.06 | 0.00 | 0.06 | 0.00 | 0.04 | 0.00 | 3.05 | 0.00 |  |
|  | 8 |  |  | 0.38 | 0.00 | 0.22 | 0.00 | 0.10 | 0.00 | 5.76 | 8.14 | 0.17 | 0.00 | 0.17 | 0.00 | 0.15 | 0.00 | 3.48 | 0.00 |  |
|  | 12 |  |  | 1.05 | 0.00 | 0.54 | 0.00 | 0.38 | 0.00 | 7.18 | 10.15 | 0.18 | 0.00 | 0.18 | 0.00 | 0.16 | 0.00 | 2.46 | 0.00 |  |
|  |  |  |  |  |  |  |  |  |  |  |  |  |  |  |  |  |  |  |  |  |
| Recipient immune cells - PB | 4 |  |  | 21.63 | 30.59 | 14.66 | 20.73 | 5.18 | 7.33 | 7.60 | 10.74 | 0.09 | 0.00 | 0.09 | 0.00 | 0.07 | 0.00 | 3.93 | 5.56 |  |
|  | 8 |  |  | 25.76 | 36.42 | 16.88 | 23.86 | 7.64 | 10.80 | 6.06 | 8.56 | 0.38 | 0.00 | 0.37 | 0.00 | 0.34 | 0.00 | 10.30 | 14.56 |  |
|  | 12 |  |  | 17.63 | 24.93 | 11.93 | 16.87 | 5.17 | 7.30 | 5.35 | 7.56 | 0.14 | 0.00 | 0.14 | 0.00 | 0.10 | 0.00 | 17.30 | 24.47 |  |

**Supplemental data table S4b:** Calculated raw data representing immune profile of DC control mIUT donor cells, maternal immune cells (MMc) and recipient cells in BM and PB. Data represents mean ± SD, analysed by two-way ANOVA with Tukey’s multiple comparisons test. Representative bar graphs are displayed in figure 3a-f.

|  |  | **Total DCC** | | **CD3** | | **CD4** | | **CD8** | | **CD19** | | **cDC** | | **cDC1** | | **cDC2** | | **NK1.1** | | |
| --- | --- | --- | --- | --- | --- | --- | --- | --- | --- | --- | --- | --- | --- | --- | --- | --- | --- | --- | --- | --- |
|  | Weeks | Mean | SD | Mean | SD | Mean | SD | Mean | SD | Mean | SD | Mean | SD | Mean | SD | Mean | SD | Mean | SD |  |
| Donor immune cells - BM | 0 | 0.90 | 0.00 | 62.21 | 0.00 | 9.45 | 0.00 | 48.53 | 0.00 | 9.77 | 0.00 | 1.02 | 0.00 | 0.27 | 0.00 | 0.40 | 0.00 | 1.30 | 0.00 |  |
|  | 4 | 0.01 | 0.00 | 22.22 | 0.00 | 22.22 | 0.00 | 0.00 | 0.00 | 33.33 | 0.00 | 0.00 | 0.00 | 0.00 | 0.00 | 0.00 | 0.00 | 0.00 | 0.00 |  |
|  | 8 | 0.01 | 0.01 | 0.00 | 0.00 | 0.00 | 0.00 | 0.00 | 0.00 | 0.00 | 0.00 | 0.00 | 0.00 | 0.00 | 0.00 | 0.00 | 0.00 | 0.00 | 0.00 |  |
|  | 12 | 0.01 | 0.01 | 0.00 | 0.00 | 0.00 | 0.00 | 0.00 | 0.00 | 0.00 | 0.00 | 0.00 | 0.00 | 0.00 | 0.00 | 0.00 | 0.00 | 0.00 | 0.00 |  |
|  |  |  |  |  |  |  |  |  |  |  |  |  |  |  |  |  |  |  |  |  |
| Donor immune cells - PB | 4 | 11.14 | 0.00 | 71.97 | 0.00 | 4.71 | 0.00 | 63.90 | 0.00 | 1.80 | 0.00 | 0.91 | 0.00 | 0.37 | 0.00 | 0.35 | 0.00 | 8.47 | 0.00 |  |
|  | 8 | 0.10 | 0.00 | 79.17 | 0.00 | 0.00 | 0.00 | 75.00 | 0.00 | 0.00 | 0.00 | 0.00 | 0.00 | 0.00 | 0.00 | 0.00 | 0.00 | 4.14 | 0.00 |  |
|  | 12 | 0.00 | 0.00 | 50.00 | 0.00 | 0.00 | 0.00 | 0.00 | 0.00 | 0.00 | 0.00 | 0.00 | 0.00 | 0.00 | 0.00 | 0.00 | 0.00 | 0.00 | 0.00 |  |
|  |  |  |  |  |  |  |  |  |  |  |  |  |  |  |  |  |  |  |  |  |
|  |  | **Total MMc** | | **CD3** | | **CD4** | | **CD8** | | **CD19** | | **cDC** | | **cDC1** | | **cDC2** | | **NK1.1** | | |
|  | Weeks | Mean | SD | Mean | SD | Mean | SD | Mean | SD | Mean | SD | Mean | SD | Mean | SD | Mean | SD | Mean | SD |  |
| MMc - BM | 0 | 1.68 | 2.38 | 2.63 | 0.00 | 0.70 | 0.00 | 0.88 | 0.00 | 0.00 | 0.00 | 0.00 | 0.00 | 0.00 | 0.00 | 0.00 | 0.00 | 0.18 | 0.00 |  |
|  | 4 | 0.02 | 0.03 | 0.00 | 0.00 | 0.00 | 0.00 | 0.00 | 0.00 | 8.11 | 11.47 | 0.00 | 0.00 | 0.00 | 0.00 | 0.00 | 0.00 | 2.70 | 0.00 |  |
|  | 8 | 0.02 | 0.03 | 5.41 | 0.00 | 0.00 | 0.00 | 5.41 | 0.00 | 24.33 | 34.40 | 0.03 | 0.00 | 0.03 | 0.00 | 0.00 | 0.00 | 10.81 | 0.00 |  |
|  | 12 | 0.00 | 0.00 | 0.00 | 0.00 | 0.00 | 0.00 | 0.00 | 0.00 | 0.00 | 0.00 | 0.00 | 0.00 | 0.00 | 0.00 | 0.00 | 0.00 | 0.00 | 0.00 |  |
|  |  |  |  |  |  |  |  |  |  |  |  |  |  |  |  |  |  |  |  |  |
| MMc - PB | 4 | 0.07 | 0.10 | 9.09 | 0.00 | 0.00 | 0.00 | 0.00 | 0.00 | 0.00 | 0.00 | 0.00 | 0.00 | 0.00 | 0.00 | 0.00 | 0.00 | 0.00 | 0.00 |  |
|  | 8 | 0.14 | 0.20 | 1.54 | 0.00 | 0.00 | 0.00 | 0.00 | 0.00 | 9.23 | 0.00 | 0.00 | 0.00 | 0.00 | 0.00 | 0.00 | 0.00 | 38.46 | 0.00 |  |
|  | 12 | 0.32 | 0.45 | 5.67 | 0.00 | 2.09 | 0.00 | 1.34 | 0.00 | 11.34 | 0.00 | 0.00 | 0.00 | 0.00 | 0.00 | 0.00 | 0.00 | 20.15 | 0.00 |  |

|  |  |  | | **CD3** | | **CD4** | | **CD8** | | **CD19** | | **cDC** | | **cDC1** | | **cDC2** | | **NK1.1** | |
| --- | --- | --- | --- | --- | --- | --- | --- | --- | --- | --- | --- | --- | --- | --- | --- | --- | --- | --- | --- |
|  | Weeks |  |  | Mean | SD | Mean | SD | Mean | SD | Mean | SD | Mean | SD | Mean | SD | Mean | SD | Mean | SD |
| Recipient immune cells - BM | 0 |  |  | 2.76 | 3.90 | 2.54 | 0.00 | 0.92 | 1.30 | 22.56 | 31.90 | 0.27 | 0.00 | 0.04 | 0.00 | 0.10 | 0.00 | 0.22 | 0.30 |
|  | 4 |  |  | 0.98 | 1.39 | 0.63 | 0.00 | 0.48 | 0.68 | 47.20 | 66.75 | 0.17 | 0.00 | 0.16 | 0.00 | 0.13 | 0.00 | 0.28 | 0.39 |
|  | 8 |  |  | 0.88 | 1.24 | 0.50 | 0.00 | 0.39 | 0.54 | 46.67 | 65.99 | 0.15 | 0.00 | 0.15 | 0.00 | 0.12 | 0.00 | 0.50 | 0.71 |
|  | 12 |  |  |  |  |  |  |  |  |  |  |  |  |  |  |  |  |  |  |
|  |  |  |  |  |  |  |  |  |  |  |  |  |  |  |  |  |  |  |  |
| Recipient immune cells - PB | 4 |  |  | 1.45 | 2.05 | 0.43 | 0.60 | 0.56 | 0.78 | 39.34 | 55.63 | 0.42 | 0.00 | 0.16 | 0.00 | 0.14 | 0.00 | 4.63 | 0.00 |
|  | 8 |  |  | 33.07 | 46.76 | 18.17 | 25.70 | 14.18 | 20.05 | 7.86 | 11.12 | 0.09 | 0.00 | 0.09 | 0.00 | 0.08 | 0.00 | 12.00 | 0.00 |
|  | 12 |  |  | 21.52 | 30.43 | 13.44 | 19.01 | 7.25 | 10.25 | 19.91 | 28.15 | 0.41 | 0.00 | 0.40 | 0.00 | 0.36 | 0.00 | 10.99 | 0.00 |

**Supplemental data table S5a:** Calculated raw data representing immune profile of DC-depleted aIUT donor cells, maternal immune cells (MMc) and recipient cells in BM and PB. Data represents mean ± SD, analysed by two-way ANOVA with Tukey’s multiple comparisons test. Representative bar graphs are displayed in figure 4a-f.

|  |  | **Total DCC** | | **CD3** | | **CD4** | | **CD8** | | **CD19** | | **cDC** | | **cDC1** | | **cDC2** | | **NK1.1** | |
| --- | --- | --- | --- | --- | --- | --- | --- | --- | --- | --- | --- | --- | --- | --- | --- | --- | --- | --- | --- |
|  | Weeks | Mean | SD | Mean | SD | Mean | SD | Mean | SD | Mean | SD | Mean | SD | Mean | SD | Mean | SD | Mean | SD |
| Donor immune cells - in BM | 0 | 0.87 | 0.46 | 25.01 | 19.99 | 12.69 | 13.45 | 4.70 | 0.25 | 0.85 | 0.37 | 4.49 | 3.06 | 3.35 | 0.04 | 4.21 | 0.76 | 5.42 | 0.17 |
|  | 4 | 0.51 | 0.00 | 13.79 | 19.50 | 0.13 | 0.18 | 6.48 | 9.16 | 1.70 | 2.40 | 0.00 | 0.00 | 0.00 | 0.00 | 0.00 | 0.43 | 1.60 | 1.65 |
|  | 8 | 0.25 | 0.00 | 17.82 | 25.20 | 2.43 | 3.43 | 4.91 | 6.94 | 3.15 | 4.45 | 0.00 | 0.00 | 0.00 | 0.00 | 0.00 | 0.00 | 1.45 | 0.00 |
|  | 12 | 0.04 | 0.01 | 32.72 | 4.94 | 12.75 | 13.67 | 5.34 | 3.88 | 12.04 | 1.26 | 1.33 | 1.88 | 0.73 | 0.07 | 1.09 | 0.07 | 2.07 | 0.17 |
|  |  |  |  |  |  |  |  |  |  |  |  |  |  |  |  |  |  |  |  |
| Donor immune cells - in PB | 4 | 0.77 | 0.00 | 64.29 | 0.00 | 16.43 | 0.00 | 23.57 | 0.00 | 2.86 | 0.00 | 0.29 | 0.41 | 0.29 | 0.41 | 0.29 | 0.41 | 0.00 | 0.00 |
|  | 8 | 0.95 | 0.00 | 64.14 | 0.00 | 21.21 | 0.00 | 14.65 | 0.00 | 1.52 | 0.00 | 1.39 | 0.00 | 1.39 | 0.00 | 1.39 | 0.00 | 0.51 | 0.00 |
|  | 12 | 0.05 | 0.04 | 44.29 | 21.62 | 32.47 | 21.78 | 5.67 | 5.01 | 12.38 | 3.97 | 2.26 | 0.27 | 1.05 | 1.49 | 1.92 | 0.76 | 5.38 | 1.58 |
|  |  |  |  |  |  |  |  |  |  |  |  |  |  |  |  |  |  |  |  |
|  |  | **Total MMc** | | **CD3** | | **CD4** | | **CD8** | | **CD19** | | **cDC** | | **cDC1** | | **cDC2** | | **NK1.1** | |
|  | Weeks | Mean | SD | Mean | SD | Mean | SD | Mean | SD | Mean | SD | Mean | SD | Mean | SD | Mean | SD | Mean | SD |
| MMc - BM | 0 | 1.02 | 1.42 | 5.07 | 0.09 | 3.35 | 2.52 | 0.08 | 0.11 | 0.02 | 0.02 | 0.00 | 0.00 | 0.02 | 0.00 | 0.09 | 0.00 | 13.27 | 2.99 |
|  | 4 | 0.06 | 0.06 | 0.05 | 0.07 | 0.05 | 0.07 | 0.10 | 0.00 | 0.10 | 0.00 | 0.00 | 0.00 | 0.00 | 0.00 | 0.00 | 0.00 | 7.20 | 10.03 |
|  | 8 | 0.01 | 0.00 | 25.05 | 35.28 | 25.05 | 35.28 | 0.10 | 0.00 | 0.10 | 0.00 | 0.00 | 0.00 | 0.00 | 0.00 | 0.00 | 0.00 | 0.05 | 0.07 |
|  | 12 | 1.37 | 0.47 | 0.03 | 0.02 | 0.02 | 0.03 | 0.00 | 0.00 | 0.50 | 0.23 | 0.03 | 0.00 | 0.01 | 0.00 | 0.00 | 0.00 | 10.82 | 0.61 |
|  |  |  |  |  |  |  |  |  |  |  |  |  |  |  |  |  |  |  |  |
| MMc - PB | 4 | 0.06 | 0.06 | 0.05 | 0.07 | 0.05 | 0.07 | 0.05 | 0.07 | 0.05 | 0.07 | 0.00 | 0.00 | 0.00 | 0.00 | 0.00 | 0.00 | 0.05 | 0.07 |
|  | 8 | 0.06 | 0.06 | 0.05 | 0.07 | 0.05 | 0.07 | 0.05 | 0.07 | 0.05 | 0.07 | 0.00 | 0.00 | 0.00 | 0.00 | 0.00 | 0.00 | 0.05 | 0.07 |
|  | 12 | 0.09 | 0.09 | 2.37 | 3.34 | 0.94 | 1.32 | 0.58 | 0.81 | 3.00 | 2.62 | 0.11 | 0.00 | 0.00 | 0.00 | 0.00 | 0.00 | 31.12 | 16.41 |

|  |  |  | | **CD3** | | **CD4** | | **CD8** | | **CD19** | | **cDC** | | **cDC1** | | **cDC2** | | **NK1.1** | |
| --- | --- | --- | --- | --- | --- | --- | --- | --- | --- | --- | --- | --- | --- | --- | --- | --- | --- | --- | --- |
|  | Weeks |  |  | Mean | SD | Mean | SD | Mean | SD | Mean | SD | Mean | SD | Mean | SD | Mean | SD | Mean | SD |
| Recipient immune cells - BM | 0 |  |  | 7.57 | 8.15 | 4.38 | 4.41 | 1.63 | 2.08 | 8.41 | 2.46 | 0.62 | 0.10 | 0.52 | 0.09 | 0.31 | 0.06 | 6.20 | 3.14 |
|  | 4 |  |  | 0.24 | 0.20 | 0.14 | 0.06 | 0.11 | 0.01 | 3.37 | 4.62 | 0.09 | 0.00 | 0.04 | 0.00 | 0.06 | 0.00 | 0.87 | 1.08 |
|  | 8 |  |  | 3.32 | 4.55 | 1.00 | 1.27 | 1.76 | 2.35 | 9.14 | 12.78 | 0.24 | 0.00 | 0.01 | 0.00 | 0.18 | 0.00 | 0.83 | 1.03 |
|  | 12 |  |  | 0.67 | 0.23 | 0.29 | 0.04 | 0.23 | 0.11 | 19.69 | 3.09 | 0.25 | 0.11 | 0.14 | 0.13 | 0.21 | 0.11 | 4.43 | 2.57 |
|  |  |  |  |  |  |  |  |  |  |  |  |  |  |  |  |  |  |  |  |
| Recipient immune cells - PB | 4 |  |  | 18.77 | 26.40 | 10.87 | 15.23 | 6.79 | 9.45 | 5.14 | 7.13 | 0.46 | 0.00 | 0.15 | 0.00 | 0.41 | 0.00 | 4.43 | 6.12 |
|  | 8 |  |  | 28.75 | 40.51 | 17.32 | 24.35 | 10.75 | 15.06 | 8.14 | 11.36 | 0.35 | 0.00 | 0.03 | 0.00 | 0.30 | 0.00 | 9.19 | 12.86 |
|  | 12 |  |  | 38.67 | 5.95 | 22.65 | 3.75 | 14.57 | 2.40 | 25.28 | 3.16 | 0.45 | 0.14 | 0.19 | 0.15 | 0.38 | 0.13 | 14.68 | 5.17 |

**Supplemental data table S5b:** Calculated raw data representing immune profile of DC control aIUT donor cells, maternal immune cells (MMc) and recipient cells in BM and PB. Data represents mean ± SD, analysed by two-way ANOVA with Tukey’s multiple comparisons test. Representative bar graphs are displayed in figure 4a-f.

|  |  | **Total DCC** | | **CD3** | | **CD4** | | **CD8** | | **CD19** | | **cDC** | | **cDC1** | | **cDC2** | | **NK1.1** | |
| --- | --- | --- | --- | --- | --- | --- | --- | --- | --- | --- | --- | --- | --- | --- | --- | --- | --- | --- | --- |
|  | Weeks | Mean | SD | Mean | SD | Mean | SD | Mean | SD | Mean | SD | Mean | SD | Mean | SD | Mean | SD | Mean | SD |
| Donor immune cells - in BM | 0 | 0.22 | 0.28 | 34.58 | 22.20 | 18.41 | 13.12 | 4.05 | 0.93 | 10.63 | 12.93 | 2.54 | 0.52 | 0.69 | 0.04 | 1.99 | 0.76 | 1.81 | 0.17 |
|  | 4 | 0.02 | 0.01 | 28.21 | 16.86 | 2.15 | 0.25 | 13.92 | 14.74 | 14.09 | 9.68 | 0.30 | 0.43 | 0.00 | 0.00 | 0.30 | 0.43 | 1.17 | 1.65 |
|  | 8 | 0.03 | 0.00 | 38.34 | 0.00 | 21.24 | 0.00 | 1.04 | 0.00 | 30.05 | 0.00 | 0.00 | 0.00 | 0.00 | 0.00 | 0.00 | 0.00 | 0.00 | 0.00 |
|  | 12 | 0.06 | 0.02 | 19.14 | 1.42 | 8.57 | 8.56 | 6.21 | 5.47 | 10.64 | 4.29 | 0.49 | 0.23 | 0.39 | 0.07 | 0.39 | 0.07 | 3.00 | 0.17 |
|  |  |  |  |  |  |  |  |  |  |  |  |  |  |  |  |  |  |  |  |
| Donor immune cells - in PB | 4 | 0.04 | 0.03 | 26.11 | 24.13 | 19.31 | 19.49 | 3.76 | 2.83 | 18.05 | 14.16 | 0.18 | 0.26 | 0.15 | 0.22 | 0.18 | 0.26 | 2.77 | 3.21 |
|  | 8 | 0.05 | 0.00 | 71.01 | 0.00 | 61.35 | 0.00 | 2.42 | 0.00 | 8.21 | 0.00 | 0.00 | 0.00 | 0.00 | 0.00 | 0.00 | 0.00 | 0.97 | 0.00 |
|  | 12 | 0.07 | 0.01 | 74.61 | 1.29 | 57.85 | 8.96 | 5.42 | 5.47 | 7.40 | 0.62 | 0.39 | 0.00 | 0.19 | 0.00 | 0.19 | 0.00 | 0.93 | 0.23 |
|  |  |  |  |  |  |  |  |  |  |  |  |  |  |  |  |  |  |  |  |
|  |  | **Total MMc** | | **CD3** | | **CD4** | | **CD8** | | **CD19** | | **cDC** | | **cDC1** | | **cDC2** | | **NK1.1** | |
|  | Weeks | Mean | SD | Mean | SD | Mean | SD | Mean | SD | Mean | SD | Mean | SD | Mean | SD | Mean | SD | Mean | SD |
| MMc - BM | 0 | 1.15 | 1.22 | 2.26 | 0.52 | 1.07 | 0.13 | 0.01 | 0.01 | 0.08 | 0.10 | 0.00 | 0.00 | 0.00 | 0.00 | 0.00 | 0.00 | 2.47 | 2.33 |
|  | 4 | 0.92 | 1.24 | 0.02 | 0.02 | 0.01 | 0.01 | 0.01 | 0.01 | 0.04 | 0.05 | 0.00 | 0.00 | 0.00 | 0.00 | 0.00 | 0.00 | 3.37 | 3.59 |
|  | 8 | 2.59 | 0.00 | 0.25 | 0.00 | 0.06 | 0.00 | 0.15 | 0.00 | 2.45 | 0.00 | 0.02 | 0.00 | 0.00 | 0.00 | 0.02 | 0.00 | 2.91 | 0.00 |
|  | 12 | 0.91 | 0.58 | 0.05 | 0.03 | 0.03 | 0.04 | 0.02 | 0.02 | 0.42 | 0.36 | 0.04 | 0.01 | 0.01 | 0.01 | 0.02 | 0.00 | 23.11 | 12.48 |
|  |  |  |  |  |  |  |  |  |  |  |  |  |  |  |  |  |  |  |  |
| MMc - PB | 4 | 0.06 | 0.01 | 1.63 | 1.37 | 0.15 | 0.21 | 0.91 | 0.35 | 1.22 | 0.33 | 0.00 | 0.00 | 0.00 | 0.00 | 0.00 | 0.00 | 9.47 | 1.73 |
|  | 8 | 0.16 | 0.00 | 6.81 | 0.00 | 1.78 | 0.00 | 4.05 | 0.00 | 24.80 | 0.00 | 0.00 | 0.00 | 0.00 | 0.00 | 0.00 | 0.00 | 34.85 | 0.00 |
|  | 12 | 0.10 | 0.03 | 1.64 | 1.75 | 0.30 | 0.00 | 2.41 | 0.00 | 8.87 | 1.24 | 0.25 | 0.00 | 0.16 | 0.00 | 0.08 | 0.00 | 45.37 | 7.93 |

|  |  |  | | **CD3** | | **CD4** | | **CD8** | | **CD19** | | **cDC** | | **cDC1** | | **cDC2** | | **NK1.1** | |
| --- | --- | --- | --- | --- | --- | --- | --- | --- | --- | --- | --- | --- | --- | --- | --- | --- | --- | --- | --- |
|  | Weeks |  |  | Mean | SD | Mean | SD | Mean | SD | Mean | SD | Mean | SD | Mean | SD | Mean | SD | Mean | SD |
| Recipient immune cells - BM | 0 |  |  | 3.66 | 2.04 | 1.86 | 0.83 | 0.79 | 0.25 | 28.45 | 23.56 | 0.29 | 0.03 | 0.08 | 0.02 | 0.13 | 0.01 | 2.32 | 0.08 |
|  | 4 |  |  | 0.85 | 0.44 | 0.38 | 0.19 | 0.34 | 0.21 | 15.47 | 0.28 | 0.11 | 0.03 | 0.09 | 0.02 | 0.09 | 0.02 | 1.27 | 0.16 |
|  | 8 |  |  | 1.00 | 0.00 | 0.56 | 0.00 | 0.32 | 0.00 | 48.98 | 0.00 | 0.18 | 0.00 | 0.14 | 0.00 | 0.15 | 0.00 | 1.80 | 0.00 |
|  | 12 |  |  | 0.75 | 0.16 | 0.39 | 0.05 | 0.28 | 0.10 | 24.10 | 9.76 | 0.24 | 0.01 | 0.21 | 0.01 | 0.20 | 0.01 | 3.00 | 2.08 |
|  |  |  |  |  |  |  |  |  |  |  |  |  |  |  |  |  |  |  |  |
| Recipient immune cells - PB | 4 |  |  | 34.34 | 7.42 | 22.84 | 4.87 | 10.57 | 2.66 | 37.78 | 7.09 | 0.65 | 0.19 | 0.61 | 0.18 | 0.56 | 0.14 | 6.17 | 0.03 |
|  | 8 |  |  | 24.55 | 34.72 | 16.21 | 22.92 | 6.75 | 9.55 | 13.21 | 18.67 | 0.27 | 0.00 | 0.24 | 0.00 | 0.23 | 0.00 | 6.01 | 8.49 |
|  | 12 |  |  | 30.35 | 4.55 | 19.73 | 2.83 | 9.70 | 1.89 | 37.66 | 4.26 | 0.39 | 0.02 | 0.32 | 0.00 | 0.30 | 0.01 | 14.76 | 1.06 |
